# The Simplest Amino‐borane H_2_B=NH_2_ Trapped on a Rhodium Dimer: Pre‐Catalysts for Amine–Borane Dehydropolymerization

**DOI:** 10.1002/anie.201600898

**Published:** 2016-04-21

**Authors:** Amit Kumar, Nicholas A. Beattie, Sebastian D. Pike, Stuart A. Macgregor, Andrew S. Weller

**Affiliations:** ^1^ Department of Chemistry, Chemistry Research LaboratoriesUniversity of OxfordMansfield RoadOxfordOX1 3TAUK; ^2^ Institute of Chemical SciencesHeriot Watt UniversityEdinburghEH14 4ASUK

**Keywords:** amino-borane, dehydrocoupling, DFT, catalytic mechanisms, rhodium dimers

## Abstract

The μ‐amino–borane complexes [Rh_2_(L^R^)_2_(μ‐H)(μ‐H_2_B=NHR′)][BAr^F^_4_] (L^R^=R_2_P(CH_2_)_3_PR_2_; R=Ph, ^i^Pr; R′=H, Me) form by addition of H_3_B⋅NMeR′H_2_ to [Rh(L^R^)(η^6^‐C_6_H_5_F)][BAr^F^_4_]. DFT calculations demonstrate that the amino–borane interacts with the Rh centers through strong Rh‐H and Rh‐B interactions. Mechanistic investigations show that these dimers can form by a boronium‐mediated route, and are pre‐catalysts for amine‐borane dehydropolymerization, suggesting a possible role for bimetallic motifs in catalysis.

Polyamino‐boranes ([H_2_BNRH]_*n*_) are potentially exciting new materials that are isoelectronic with technologically pervasive polyolefins, but are chemically distinct because of (δ−)HB−NH(δ+) polarization. They are formed by the dehydropolymerization of amine‐boranes (H_3_B⋅NRH_2_; R=H or Me, for example; Scheme 1 A),^1^ and metal‐catalyzed routes to polyamino‐boranes offer the potential for fine control over molecular weight and polymer stereochemistry. There is recent evidence that these processes occur at a metal center in which the catalyst needs to perform two roles: 1) formal dehydrogenation of amine‐borane to form a latent source of amino‐borane (H_2_B=NRH), and 2) subsequent B−N bond formation.^2^, ^3^, ^4^, ^5^, ^6^ For some systems a coordination/insertion mechanism is proposed, although the precise structure of the propagating species is currently unresolved (Scheme 1 B).^3^, ^5^, ^6^ This is in contrast to olefin polymerization, in which the feedstock (for example, ethene or propene) is already unsaturated, and the active species and propagating mechanisms are well‐defined.^7^ A clearer understanding of how the catalyst dehydrogenates amine‐borane, traps intermediate amino‐boranes, and promotes B−N bond‐formation, is central to harnessing the full potential of systems that ultimately deliver new well–defined B−N polymeric materials on a useful scale.

**Scheme 1:**
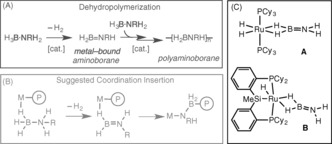
A) Amine‐borane dehydropolymerization; B) a suggested coordination/insertion mechanism, P=polymer chain; C) examples of H_2_B=NH_2_ coordinated to a metal center.

Unlike ethene (H_2_C=CH_2_), which is stable under ambient conditions, the isoelectronic amino‐borane (H_2_B=NH_2_) has only been prepared in low temperature matrices and oligomerizes above −150 °C.^2^, ^8^ Adding steric bulk to the nitrogen atom increases stability, so that, for example, H_2_B=NMeH^9^ or H_2_B=N^t^BuH^10^ can be observed as transient species using in situ NMR spectroscopy before they also oligomerize. There are two examples where unstable H_2_B=NH_2_ can be trapped by coordination to a single metal center. These originate after dehydrogenation of a putative σ‐ammonia borane^11^ complex, forming Ru(PCy_3_)_2_(H)_2_(η^2^‐H_2_B=NH_2_) **A** 12 and (Cy‐PSiP)‐Ru(H)(η^2^‐H_2_B=NH_2_) **B**, Cy‐PSiP=κ^3^‐(Cy_2_PC_6_H_4_)_2_SiMe).^13^

We now report that H_2_B=NH_2_ can be trapped by a bimetallic [Rh_2_(R_2_PCH_2_CH_2_CH_2_PR_2_)_2_]^2+^ fragment to give a novel bridging amino‐borane bonding motif. We provide mechanistic evidence for formation of the complex from a monometallic precursor, and show that such dimeric amino‐borane species may be important in dehydropolymerization pathways. This report builds upon previous observations that indirectly implicate bimetallic motifs during catalysis.^14^, ^15^, ^16^

Addition of a slight excess of H_3_B⋅NH_3_ to a [D_8_]THF solution of [Rh(L^Ph^)(η^6^‐C_6_H_5_F)][BAr^F^_4_] **1** (L^Ph^=Ph_2_P(CH_2_)_3_PPh_2_, Ar^F^=3,5‐(CF_3_)_2_C_6_H_3_) resulted in the rapid formation of a bimetallic monocation, which was identified by NMR spectroscopy, electrospray ionization mass spectrometry (ESI‐MS), and single‐crystal X‐ray diffraction, as [Rh_2_(L^Ph^)_2_(μ‐H)(μ‐H_2_B=NH_2_)][BAr^F^_4_] **3**. One equivalent of the boronium^9^, ^17^, ^18^, ^19^, ^20^ cation [THF⋅BH_2_⋅NH_3_][BAr^F^_4_] was also formed (*δ*(^11^B) 0.5 (t), *J*_BH_=108 Hz; lit.^19^ [Et_2_O⋅BH_2_⋅NH_3_][BAr^F^_4_] *δ*(^11^B) 0.2, *J*_BH_=125 Hz).

In situ solution NMR data for **3** show a signal at *δ*(^11^B) 51.5, a single ^31^P environment (*δ*(^31^P) 18.2, *J*_RhP_=142 Hz), and a broad peak at *δ*(^1^H) −7.45 (integral ca. 3H relative to the phenyl groups). ESI‐MS shows a mono‐cation at *m*/*z=*1060.16 (calcd 1060.16) with the correct isotope pattern. Crystallization (THF/pentane/‐18 °C) gave a small number of crystals, for which a single‐crystal X‐ray diffraction study showed a H_2_B=NH_2_ unit bridging a {(Rh_2_(L^Ph^)_2_(μ‐H)} unit (Supporting Information, Figure S21). However, insufficient material was obtained upon which to collect reliable NMR data. Complex **3** is unstable in solution at room temperature, decomposing after four hours to give a mixture in which [Rh(L^Ph^)(THF)_2_][BAr^F^_4_] **6** was present in approximately 30 % yield.^21^ To put the structure and spectroscopic data on a firm footing, the equivalent reaction using the ^i^Pr‐substituted chelating phosphine gave complex **4**, [Rh_2_(L^iPr^)_2_(μ‐H)(μ‐H_2_B=NH_2_)][BAr^F^_4_], and **5** (Scheme 2). This reaction was slower than that observed for L^Ph^. Complex **4** can also be isolated in 78 % yield as orange crystalline material using an alternative route (see below, Scheme 5). In the absence of H_3_B⋅NH_3_, complex **4** is stable for at least two days in [D_8_]THF solution. However, when formed in situ **4** decomposes over 24 hrs into a mixture of products, one of which can be characterized as [Rh_2_(L^iPr^)_2_(H)_2_(μ‐H)_3_][BAr^F^_4_].^22^ The room temperature solution NMR data obtained for **4** are very similar to those for **3**: *δ*(^11^B) 51.1; *δ*(^31^P) 40.8, *J*_RhP_=142 Hz; *δ*(^1^H) −8.64 (3 H, broad). Progressive cooling to 180 K splits the high field hydride resonance into two signals, in a 2:1 ratio; while two ^31^P environments were also observed, suggesting a fluxional process at room temperature. An Eyring plot yields the activation data: Δ*H* ^≠^=31.1±1.3 kJ mol^−1^, Δ*S* ^≠^= −27±1 J K^−1^ mol^−1^, Δ*G*(298 K)^≠^=39.2±1.6 kJ mol^−1^; where the negative entropy of activation suggests an intramolecular process (Supporting Information, Figures S2–3).

**Scheme 2:**
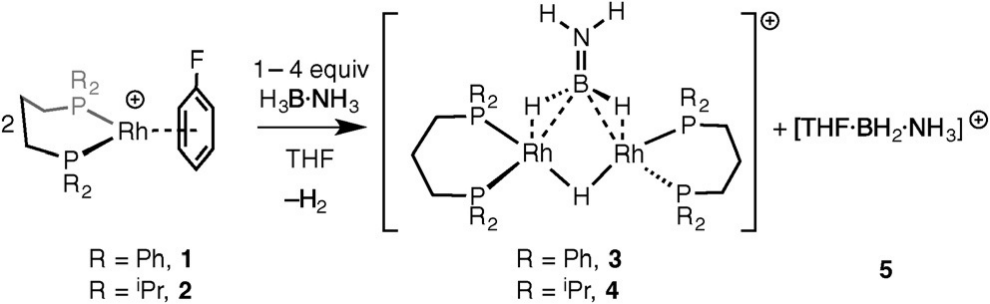
Formation of amino‐borane coordinated dimers **3** and **4**. [BAr^F^_4_]^−^ anions are not shown.

The solid‐state structure of complex **4** is shown in Figure 1 A. A dimeric Rh_2_ unit is accompanied by one [BAr^F^_4_]^−^ anion, confirming that it is a mono‐cation. Two {Rh(L^iPr^)}^+^ fragments are bridged by a hydride and a H_2_B=NH_2_ unit. The B−N distance (1.377(6) Å) is consistent with a significant B−N π‐interaction, and is similar to that measured in **A** (1.396(3) Å) and **B** (1.359(8) Å), as well as the bridging borylene complex **C** (1.399(3) Å; Scheme 3).^23^ The Rh⋅⋅⋅B distances (2.070(5) and 2.055(5) Å) are similar to those found in the amino‐borane complexes **A**, **B**, and [Ir(PCy_3_)_2_(H)_2_(H_2_B=NMe_2_)][BAr^F^_4_]^24^ (spanning 1.956(2) to 2.140(13) Å), but significantly shorter than those measured in the bridging thexylborohydride complex **D** (2.330(3) Å).^25^ The hydrogen atoms were located but refined using a riding model. Within the limits of X‐ray diffraction the B−H distances suggest lengthened, but unbroken bonds (for example, 1.360 Å). The NH_2_ group is slightly twisted with respect to the BH_2_ group (24.3°; Figure 1 B). The whole H_2_B=NH_2_ fragment lies 54.1° from the Rh‐Rh vector so as to accommodate appropriate overlap between the B−H bonds and the two rhodium centers. These are best described as being two distorted square planes (for example, P1/P2/H3/H1) twisted with respect to one another by 102° (Figure 1 C). This motif, which is similar to that observed for **D**, is fully consistent with the low temperature NMR data, and are recreated well in the DFT calculated structure (Supporting Information, Figures S24–26). Each metal center in **4** is best described as Rh^I^, with no M−M bond.^26^ The end‐on {Rh_2_(μ‐H_2_B=NH_2_)} binding mode contrasts with H_2_C=CH_2_ that bridges two metal centers symmetrically using both carbon atoms, in either *μ*‐*η* ^2^:*η* ^2^ or *μ*‐*η* ^1^:*η* ^1^ bonding modes,^27^, ^28^ highlighting the differences between these isosteres.^29^

**Figure 1:**
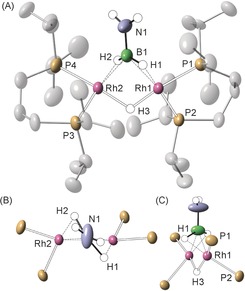
Solid‐state structure of the cationic portion of complex **4**. Displacement ellipsoids are shown at the 50 % probability level. Selected bond distances (Å) and angles (°): Rh1⋅⋅⋅Rh2, 2.7874(4); Rh1−B1, 2.070(5); Rh2−B1, 2.055(5); B1−N1 1.377(6); P1−Rh1, 2.2550(10); P2−Rh1, 2.3063(10); Rh1−H1, 1.718; Rh2−H2, 1.723; ∡plane (N1B1H1H2)/plane (N1B1Rh1Rh2), 54.1; ∡plane (Rh1P1P2)/plane (Rh2P3P4), 100.2; ∡(NH_2_)/(BH_2_) 24.3°.

**Scheme 3:**
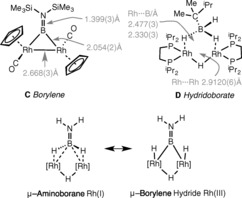
Limiting valence bond descriptions for complex **4**, and examples of bridging hydridoborate and borylene complexes. [Rh]={Rh(L^iPr^)}, charge not shown.

Surprisingly, the amino‐borane in **4** is quite strongly bound. It is only slowly displaced by excess acetonitrile (7 % in 50 min) to give a mixture of species, one of which is [Rh(L^iPr^)(NCMe)_2_][BAr^F^_4_].^22^ No reaction occurs with toluene, which might be expected to form a [Rh(L^iPr^)(η^6^‐C_6_H_5_Me)]^+^ complex if a monomeric {Rh(L^iPr^)}^+^ fragment were accessible.^30^ Addition of cyclohexene, shown to be a probe for free H_2_B=NH_2_,^2^ gave no reaction. In contrast, H_2_ rapidly reacts with **4** to form [Rh_2_(L^iPr^)_2_(H)_2_(μ‐H)_3_][BAr^F^_4_].^22^

There are two limiting forms for the structure of **4** (and quasi‐isostructural **3**): 1) a bridging amino‐borane at two Rh^I^ centers, or 2) a bridging borylene dihydride (Rh^III^), Scheme 3. The observed *δ*(^11^B) chemical shift of 51 ppm is more consistent with the former as amino‐boranes bound to one metal center show chemical shifts around 40–50 ppm,^12^, ^13^, ^24^, ^31^ while bridging borylenes^32^ are generally observed between 90 and 100 ppm.^23^, ^33^

To probe the bonding of the amino‐borane ligand in **4**, DFT calculations were used as the basis for a Quantum Theory of Atoms in Molecules (QTAIM) analysis of the total electron density. The results are presented in Figure 2 A, along with selected bond critical point (BCP) metrics. Figure 2 B provides comparative BCP data for the bridging borylene complex **C**, the hydridoborate complex **D**, and [(PPh_3_)_2_Rh(H)(μ‐H)(μ‐Cl)_2_Rh(H)(PPh_3_)_2_]^+^, **E**, a well‐defined Rh^III^ dimer with both terminal and bridging hydrides.^34^ Average data are presented for all complexes where appropriate, although the discussion will focus on the bonding around a single rhodium center (Rh1).

**Figure 2:**
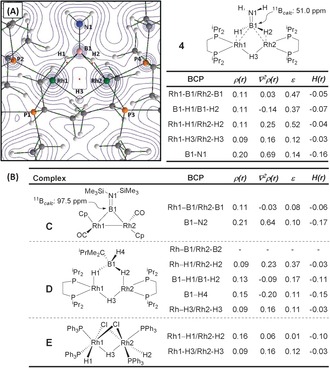
A) Contour plot of the electron density of the central part of **4** presented in the {Rh1B1Rh2} plane with projected stationary points, bond paths, bond critical points (BCP; green), and ring critical points (RCP; red); the associated table shows selected BCP metrics (a.u.; average data for indicated bonds) and computed *δ*(^11^B) chemical shifts. B) Calculated BCP metrics (a.u.; average data for indicated bonds) for comparator complexes **C** (including the computed ^11^B chemical shift), **D** and **E** (*ρ*(*r*)=electron density, ∇*ρ*(*r*)=Laplacian of electron density, *ɛ*=bond ellipticity, *H*(*r*)=local energy density). All geometries are based on the crystallographically determined heavy atom positions with hydrogen atoms optimized with the BP86 functional. For a full summary of parameters see Figures S24–27 and associated Tables in the Supporting Information.

In **4**, the {Rh1/B1/H1} moiety displays bond paths between all three centers, and these enclose a ring critical point (RCP). Thus, **4** has direct Rh1−B1 and Rh1−H1 bonding interactions, while the B1−H1 bond is also intact. Comparison with the Rh1−B1 interaction in **C** provides similar *ρ*(*r*) and *H*(*r*) values, but highlights a much reduced bond ellipticity (*ɛ*) of 0.08; this low value indicates dominant σ‐bond character, whereas the value of 0.47 in **4** reflects the asymmetry introduced by the B1‐H1 unit. In **D**, the absence of Rh‐B BCPs confirms a lack of any direct Rh‐B interaction, and this also reduces the average ellipticity of the Rh1−H1 and B1−H1 bonds. Also noticeable are the higher values of *p(r)* and *H(r)* for the terminal B1‐H4 bond in **D** compared to the bridging B‐H bonds in both that structure and, in particular, **4**, all of which is consistent with a weakening of the latter. For **E**, the Rh1‐H1 BCP has larger values for *ρ*(*r*) and *H*(*r*) than the Rh1‐H1 BCP in **4**, as well as a minimal *ɛ* value. These data indicate a terminal Rh−H σ‐bond and stress the differences in bridging character of H1 and H2 in **4**. BCP data for the Rh1−H3−Rh2 bonds in **4**, **D**, and **E** are very similar, suggesting that this moiety varies little across these three systems.

Taken together, the QTAIM analyses suggest that **4** is best described as a μ‐amino‐borane Rh^I^ species; a μ‐borylene hydride Rh^III^ formulism can certainly be ruled out in light of the intact B1−H1/B1−H2 bonds and the lack of Rh1−H1/Rh2−H2 terminal hydride character. The μ‐amino‐borane ligand in **4** interacts with the rhodium centers through stretched B−H bonds that engage in strong Rh‐H and Rh‐B interactions. Further support for this assertion comes from the computed *δ*(^11^B) chemical shifts (Figure 2) and the Pipek–Mezey localized orbitals, where a strong bonding interaction spanning all three Rh1, B1, and H1 centers was identified (see Figure 3).

**Figure 3:**
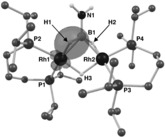
Pipek–Mezey localized orbital, highlighting the bonding interaction of the B1−H1 bond with center Rh1 (see Supporting Information, Figure S28, for details and related orbitals spanning the {Rh2B1H2} and {Rh1H3Rh2} moieties).

The mechanism of the room temperature fluxional process observed for **4** was also probed with DFT calculations and a single transition state was found to account for this process (Scheme 4). This is accessed by cleavage of one (blue) B‐H bond to give a transition state structure featuring two Rh‐H‐Rh bridging hydrides; movement of the original (red) Rh‐H‐Rh hydride into a Rh‐H‐B bridging position then completes the exchange (**4′**). Repeating this process from **4′** exchanges a second B‐H hydrogen (black) into the Rh‐H‐Rh bridging position (**4′′**). The computed free energy of activation is 55.2 kJ mol^−1^, somewhat higher than the experimental value (39.2±1.6 kJ mol^−1^) but still consistent with facile room temperature exchange.

**Scheme 4:**
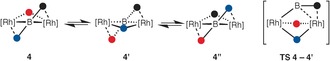
Proposed fluxional process occurring in **4** (and **3**). Hydrogen atoms shown by filled circles. See Supporting Information for DFT calculated geometries and energies.

Understanding how bimetallic species such as **3** and **4** are formed, and subsequently react, is important for delineating their role in amine‐borane dehydrocoupling. The single equivalent of boronium [THF⋅BH_2_⋅NH_3_][BAr^F^_4_] (**5**) formed indicates that a hydride abstraction route may be operating, as recently outlined by Conejero and co‐workers for the dehydrocoupling of H_3_B⋅NMe_2_H by cationic {Pt‐NHC}^+^ catalysts,^17^ as well as that occurring in cationic Ru/Ir‐systems^35^ or with B(C_6_F_5_)_3_.^19^ We reasoned that a similar process would yield **5** by B‐H activation^16^ and subsequent attack by THF (Scheme 5), alongside {Rh(L^R^)H} that would dimerize to give neutral [Rh(L^R^)H]_2_ (for example, complex **H**). Subsequent protonation^17^ by boronium **5** and elimination of H_2_ would give H_2_B=NH_2_ trapped on a rhodium dimer. To test this hypothesis, addition of **5** to the neutral dimer is required. [Rh(L^Ph^)H]_2_ is unknown, and our attempts to prepare it have not been successful. [Rh(L^iPr^)H]_2_ is a known complex, first prepared by Fryzuk in 1989,^36^ and addition of one equivalent of the known boronium salt [Et_2_O⋅BH_2_⋅NH_3_][BAr^F^_4_]^19^ to [Rh(L^iPr^)H]_2_ in Et_2_O solvent, resulted in the immediate formation of **4** and gas evolution (H_2_), which is consistent with the mechanism shown.

**Scheme 5:**
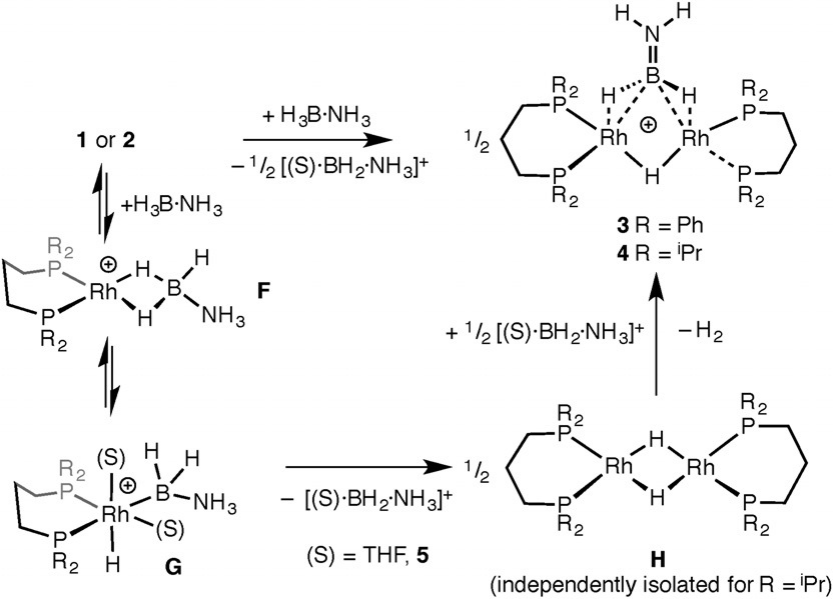
Mechanism of formation of **3** and **4** by boronium protonation of neutral dimer **H**. (S)=THF or Et_2_O. [BAr^F^_4_]^−^ anions are not shown.

A dimeric species similar to **3** was also formed when one equivalent of H_3_B⋅NMeH_2_ was added to **1** in THF solution. This was characterized by in situ NMR spectroscopy and ESI‐MS as [Rh_2_(L^Ph^)_2_(μ‐H)(μ‐H_2_B=NMeH)][BAr^F^_4_] **8**: *δ*(^1^H) −6.84; *δ*(^31^P{^1^H}) 22.2, 21.5; *δ*(^11^B) 50.6.^21^ [THF⋅BH_2_⋅NMeH_2_][BAr^F^_4_] was also formed (*δ*(^11^B) 2.8 (t), *J*_HB_=123 Hz; lit. Et_2_O adduct *δ*(^11^B, CD_2_Cl_2_) 1.7 (t), *J*_HB_=121 Hz^9^). A more complex mixture of species was formed with H_3_B⋅NMe_2_H, suggesting steric factors may be important in the formation of these aminoborane dimers, although a signal observed at *δ*(^11^B) 52.7 suggests dimer formation. Complexes **3**, **4**, and **8** presumably form via a σ‐complex [Rh(L^R^)(H_3_B⋅NRH_2_)][BAr^F^_4_], R=H (**F** Scheme 5) or Me. In THF solution, using the L^Ph^ ligand, these σ‐complexes were not observed as boronium formation and subsequent formation of **3** is fast. For L^iPr^, an intermediate σ‐complex could be observed on the way to **4**, [Rh(L^iPr^)(H_3_B⋅NH_3_)][BAr^F^_4_], presenting NMR data consistent with structure **F**.^21^ Using H_3_B⋅NMe_3_ (in which the N−H bonds are absent) [Rh(L^iPr^)(H_3_B⋅NMe_3_)][BAr^F^_4_] (**7**) was isolated and structurally characterized, confirming the in situ NMR studies (Supporting Information, Figure S23). The rapid reaction of [Et_2_O⋅BH_2_⋅NH_3_][BAr^F^_4_] with [Rh(L^iPr^)H]_2_ to form **4** suggests protonation is not slow for this system; currently we cannot determine whether B−H activation or boronium formation is the rate limiting process, although it is likely that either could be promoted by excess amine‐borane via N‐H⋅⋅⋅H‐B interactions.^37^ Calculations on the {Pt‐NHC}^+^/H_3_B⋅NMe_2_H system suggest boronium formation is rate limiting.^17^

Complex **1** (0.5 mol %, THF, 3 hrs, open system) promoted the dehydrocoupling of H_3_B⋅NH_3_ (1.2 equiv of H_2_ evolved by gas burette; Supporting Information, Figures  S4–S7) to form oligomeric species such as *B*‐(cyclotriborazanyl)amine‐borane (BCTB),^3^, ^38^ and insoluble polyamino‐borane.^3^ With more soluble H_3_B⋅NMeH_2_, polymethylamino‐borane was formed [H_2_BNMeH]_*n*_, which was isolated by precipitation from hexanes (*M_w_*=30 600 g mol^−1^, *Ð*=2.6), alongside H_2_ (1.1 equiv, gas burette). Consistent with the rapid formation of dimers such as **8** in THF, no induction period was observed (as measured by H_2_ evolution) and similar TOF values were recorded (ca. 200 hr^−1^ for 1 equiv H_2_), starting from monomeric **1** or in situ formed dimeric **8** (Scheme 6).^39^ Changing the solvent to non‐nucleophilic 1,2‐F_2_C_6_H_4_, and using **1** or in situ generated **8** as a catalyst, did not present an induction period and also revealed a faster TOF (for **8**, ca. 1000 hr^−1^ with 1 equiv of H_2_ released).^40^ Sub‐catalytic in situ experiments in this solvent^21^ show that dimer **8**, [(BH_2_)_2_NMeH(μ‐H)] and boronium [(NH_2_Me)_2_BH_2_][BAr^F^_4_] are present;^41^ the latter is suggested to arise from NMeH_2_ formed from B−N bond cleavage in H_3_B⋅NMeH_2_.^17^ Thus, it is likely that similar active species are present in THF or 1,2‐F_2_C_6_H_4_. The lack of induction period is in direct contrast to xantphos‐based rhodium catalysts, which show induction periods for H_3_B⋅NMeH_2_ dehydrocoupling in C_6_H_5_F,^5^, ^15^ suggesting that a different kinetics regime or mechanism is in operation.

**Scheme 6:**
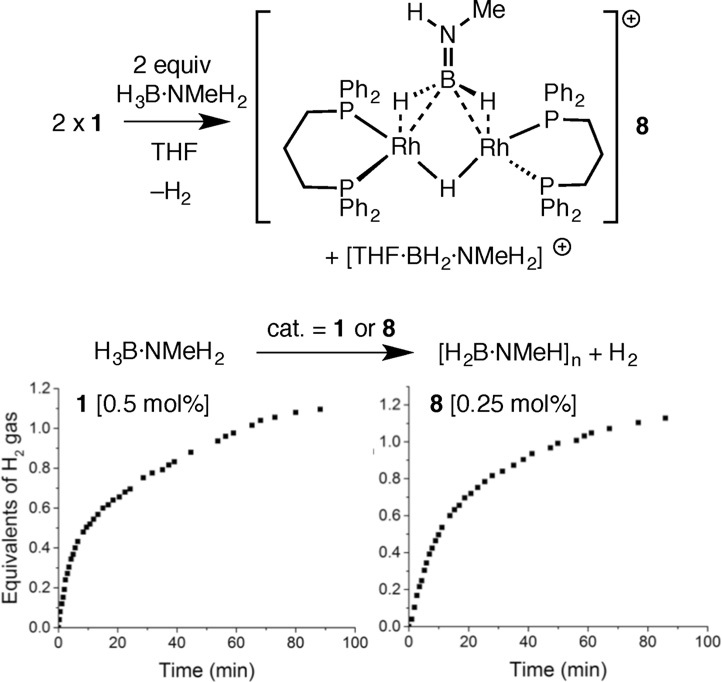
H_2_ evolution experiments using **1** or **8**, and H_3_B⋅NMeH_2_ (0.5 mol % [Rh], 0.41 m amine‐borane, THF, 298 K). [BAr^F^_4_]^−^ anions are not shown.

Determination of the resting state in catalysis was hampered by the addition of excess amine‐borane (H_3_B⋅NH_3_ or H_3_B⋅NMeH_2_) to the preformed dimeric species **3** or **4** in THF, resulting in a mixture of products that have been resistant to characterization. Turning to the pure and well‐characterized dimer **4**, initial rate measurements in a closed system (4 mol % rhodium, THF) were more informative, and a first‐order dependence for either H_3_B⋅NH_3_ or H_3_B⋅NMeH_2_, as well as catalyst **4**, were measured for the early pseudo zero‐order phase of catalysis (Supporting Information, Figures S19 and 20). Such behavior is not consistent with a rapid dimer–monomer equilibrium for which an order of [**4**]^1/2^ would be expected,^22^, ^36^, ^42^ a view supported by the stoichiometric reactions with acetonitrile or toluene (see above). Under these conditions complexes **2** or **4** do not promote full conversion of amine‐borane (for **4**, 70 % conversion of H_3_B⋅NH_3_ after 10 hrs). Informed by the sub‐catalytic experiments and H_2_ addition studies, we propose that [Rh_2_(L^iPr^)_2_(H)_2_(μ‐H)_3_][BAr^F^_4_]^22^ is formed during catalysis. Consistent with this hypothesis, isolated [Rh_2_(L^iPr^)_2_(H)_2_(μ‐H)_3_][BAr^F^_4_] is a poorer catalyst for H_3_B⋅NH_3_ dehydrocoupling in a sealed system (4 mol % [Rh], 30 % conversion after 10 hrs) than both **2** and **4**. Interestingly, degassing the closed system restarted catalysis, indicating that inhibition by the H_2_ formed during dehydrocoupling is partially reversible (Supporting Information, Figure S10). Co‐promotion of dehydrocoupling by boronium is discounted, as these studies show that isolated **4** is an active pre‐catalyst in its absence. Consistent with this statement, dehydrocoupling of H_3_B⋅NH_3_ is not catalyzed by [Et_2_O⋅BH_2_⋅NH_3_][BAr^F^_4_] under the conditions used here (0.5 mol %, THF, 298 K, 3 hrs).^19^ Overall, these observations do not let us discriminate between active catalysts derived from dimeric **4** (or **3**) or monomeric species that result from irreversible, but fast, consumption of **4** (or **3**), under the conditions of excess amine‐borane.^43^

The ambiguity surrounding mono/bimetallic catalysis has parallels with xantphos‐based amine‐borane dehydropolymerization catalysts, where P‐C activated phosphido‐bridged species are formed that are also active catalysts, in contrast to the amino‐borane‐bridged dimers observed here.^15^ Deconvoluting these systems under conditions of high amine‐borane concentration is thus a significant challenge to address if precise control over the resulting polyamino‐borane is to be achieved by metal/ligand design. Nevertheless, the observation of novel and unexpected bridging amino‐borane complexes as the first‐formed species, offers tantalizing clues as to the nature of the actual catalysts; and also suggests that boronium cations may play a more general role in amine‐borane dehydrocoupling than generally appreciated.^17^, ^19^

## Supporting information

As a service to our authors and readers, this journal provides supporting information supplied by the authors. Such materials are peer reviewed and may be re‐organized for online delivery, but are not copy‐edited or typeset. Technical support issues arising from supporting information (other than missing files) should be addressed to the authors.

Supplementary
