# Supplementary material for: The Simplest Amino‐borane H2B=NH2 Trapped on a Rhodium Dimer: Pre‐Catalysts for Amine–Borane Dehydropolymerization
Source: Angew Chem Int Ed Engl. 2016 Apr 21;55(23):6651–6. doi: 10.1002/anie.201600898 (PMC5074255; doi:10.1002/anie.201600898)
Supplement: Supplementary file 1 — Supplementary [file ANIE-55-6651-s001.pdf]

Angewandte Chemie
Eine Zeitschrift der Gesellschaft Deutscher Chemiker

Supporting Information

The Simplest Amino-borane $H_2B{=}NH_2$ Trapped on a Rhodium Dimer: Pre-Catalysts for Amine-Borane Dehydropolymerization

Amit Kumar, Nicholas A. Beattie, Sebastian D. Pike, Stuart A. Macgregor, and Andrew S. Weller**

anie_201600898_sm_miscellaneous_information.pdf

1. General experimental procedures S1
2. Synthesis of new complexes S2
3. Variable temperature NMR spectra of **4** and Eyring analysis S5
4. Stoichiometric reactions of $H_3B{\cdot}NH_3$ with **1** and **2** in THF S6
5. Reaction of 4 equivalents of $H_3B{\cdot}NH_3$ with **2** in THF S7
6. General method for the catalytic dehydrocoupling (open system) S8
7. Catalytic dehydrocoupling of $H_3B{\cdot}NH_3$ using 0.5 mol% **1** in THF S8
8. Catalytic dehydrogenation of $H_3B{\cdot}NH_3$ using 0.5 mol% $[BH_2(NH_3)(OEt_2)][BAr^F_4]$ in THF S10
9. Catalytic dehydrocoupling of $H_3B{\cdot}NH_3$ using **2** (4 mol%) or $[\{Rh(P^iPr_2(CH_2)_3P^iPr_2)\}_2(H)_2(\mu\text{-}H)_3][BAr^F_4]$ (2 mol%) in THF in a closed system S11
10. Catalytic dehydrocoupling of $H_3B{\cdot}NH_3$ using **2** (4 mol%) or **4** (2 mol%) in THF in a closed system S11
11. Reactivity of $H_3B{\cdot}NH_2Me$ with **1** in THF: Formation of **8** S13
12. Reactivity of $H_3B{\cdot}NH_2Me$ with **1** in 1,2-$F_2C_6H_4$: Formation of **8** S13
13. Catalytic dehydrocoupling of $H_3B{\cdot}NH_2Me$ using 0.5 mol% **1** in THF S14
14. Catalytic dehydrocoupling of $H_3B{\cdot}NH_2Me$ using 0.5 mol% **1** in 1,2-$F_2C_6H_4$ S16
15. Preparation (*in situ*) of **8** in THF S17
16. Catalytic dehydrocoupling of $H_3B{\cdot}NH_2Me$ using *in situ* prepared **8** (0.25 mol%) in THF S17
17. Catalytic dehydrocoupling of $H_3B{\cdot}NH_2Me$ using *in situ* prepared **8** (0.25 mol%) in 1,2-$F_2C_6H_4$ ... S18
18. Catalytic dehydrocoupling of $H_3B{\cdot}NMe_2H$ in 1,2-$F_2C_6H_4$ S19
19. Reactivity of $H_3B{\cdot}NMe_2H$ with **1** in THF: Formation of $[\{Rh(PPh_2(CH_2)_3PPh_2)\}_2(\mu\text{-}H)(\mu\text{-}BH_2NMe_2)][BAr^F_4]$ S20
20. Initial rate studies in THF in a closed system S20
21. X-ray crystallography details S22
22. Computational details and references S27
23. QTAIM results S29
24. Calculated ^{11}B NMR shifts S36
25. Pipek-Mezey localised orbitals S37
26. Cartesian coordinates (Å) and computed energies (in hartrees) for all optimised structures S38
27. References S44

1. General experimental procedures

All manipulations, unless otherwise stated, were performed under an argon atmosphere using standard Schlenk and glove-box techniques. Glassware was oven dried at 130°C overnight and flamed under vacuum prior to use. Ether, pentane and MeCN were dried using

a Grubbs type solvent purification system (MBraun SPS-800) and degassed by successive freeze-pump-thaw cycles.[1] THF was distilled from sodium benzophenone ketyl, degassed by successive freeze-pump-thaw cycles and stored over 3 Å molecular sieves. 1,2-$F_2C_6H_4$ was dried over CaH_2, vacuum distilled and stored over 3 Å molecular sieves. [Rh($P^iPr_2(CH_2)_3P^iPr_2$)(C_6H_5F)][BAr^F_4],[2] [Rh($P^iPr_2(CH_2)_3P^iPr_2$)(μ-H)]$_2$,[3] [$BH_2(NH_3)(OEt_2)$][BAr^F_4],[4] [Rh($PPh_2(CH_2)_3PPh_2$)(C_6H_5F)][BAr^F_4][5], $H_3B{\cdot}NMeH_2$[6] and [{Rh($P^iPr_2(CH_2)_3P^iPr_2$)}$_2$(H)$_2$(*μ*-H)$_3$][BAr^F_4][8] were prepared by literature methods. $H_3B{\cdot}NH_3$, $H_3B{\cdot}NMe_2H$ and $H_3B{\cdot}NMe_3$ were purchased from Aldrich and sublimed prior to use at conditions 1 x 10^{-2} Torr, 333 K (for $H_3B{\cdot}NH_3$) and 5 x 10^{-2} Torr, 298 K (twice for $H_3B{\cdot}NMe_2H$ and once for $H_3B{\cdot}NMe_3$). In C_6H_5F and 1,2-$F_2C_6H_4$, ^1H NMR spectra were referenced to the centre of the downfield solvent multiplet, δ = 7.11 and 7.07 respectively. ^{31}P and ^{11}B NMR spectra were referenced against 85% H_3PO_4 (external) and $BF_3{\cdot}OEt_2$ (external) respectively. The spectrometer was pre-locked and pre-shimmed to the solvent mixture of 0.3 ml of 1,2-$C_6H_4F_2$ and 0.1 ml of C_6D_6. Chemical shifts (δ) are quoted in ppm and coupling constants (*J*) in Hz. ESI-MS were recorded on a Bruker micrOTOF instrument interfaced with a glove-box.[7] Microanalysis was performed by Elemental Microanalysis Ltd. Initial rates were calculated from the pseudo zero-order region of the consumption of amine-boranes as measured by ^{11}B NMR spectroscopy over the first 10 minutes of catalysis. Catalytic dehydrocoupling of amine-boranes in open system was carried in a 3-necked Schlenk flask connected to an external mineral oil bubbler with tubing attached. Evolution of hydrogen gas vs. time was monitored by attaching a cannula to the Schlenk, the other end of the cannula was placed under an upturned 50 mL burette filled with water. Gel permeation chromatography (GPC) was performed on a Waters 1515 equipped with an isocratic HPLC pump. Styragel HR5E THF (4.6 x 300 mm) columns were used. THF containing 0.1% w/w [^nBu4N]Br was used as the eluent at a flow rate of 1.0 mL min^{-1}. Samples were dissolved in the eluent (2 mg mL^{-1}), left for 6 h at room temperature and filtered with an Acrodisc CR 13 mm syringe filter (with 0.2 μm PTFE membrane) before analysis. The calibration was conducted using a Polystyrene Ready-Cal kit.

2. Synthesis of new complexes

[(Rh($P^iPr_2(CH_2)_3P^iPr_2$))$_2$(μ-H)(μ-BH_2NH_2)][BAr^F_4] (4)

[Rh($P^iPr_2(CH_2)_3P^iPr_2$)(μ-H)]$_2$ (47 mg, 6.2 x 10^{-2} mmol) and [$BH_2(NH_3)(OEt_2)$][BAr^F_4] (62 mg, 6.2 x 10^{-2} mmol) were added to a Schlenk flask to which Et_2O (~3 mL) was added. Addition of Et_2O led to immediate bubbling and the resulting orange solution was stirred for 10

minutes. The orange solution was filtered, concentrated to ~1 mL and then layered with pentane at –18 ºC to yield [(Rh(P^{i}Pr$_2$(CH$_2$)$_3$P^{i}Pr$_2$))$_2$(μ-H)(μ-BH$_2$NH$_2$)][BAr$^F_4$] as orange crystals. Yield: 80 mg, 78%.

^{1}H NMR (500 MHz, d$_8$-THF): δ 7.79 (s, 8H, [BAr$^F_4$]$^-$), 7.57 (s, 4H, [BAr$^F_4$]$^-$), 4.55 (s, 2H, NH$_2$), 2.09 (br, 8H, CH), 1.25 (m, 24H, CH$_3$), 1.12 (m, 24H, CH$_3$), 0.11 (br, 12H, CH$_2$CH$_2$CH$_2$), −8.64 (s, 3H, BH$_2$ and RhH).

^{1}H NMR (500 MHz, d$_8$-THF, 180 K): δ 7.90 (s, 8H, [BAr$^F_4$]$^-$), 7.75 (s, 4H, [BAr$^F_4$]$^-$), 5.05 (s, 2H, NH$_2$), 1.38 – 0.96 (broad overlapping multiplet, 48 H, CH$_3$), 0.11 (br, 12H, CH$_2$CH$_2$CH$_2$), −8.16 (br d, $^2J_{HP}$ = 56, 2H, BH$_2$), −9.02 (br, 1H, RhH), CH signals are obscured by THF signals. The resonances (chemical shifts and respective couplings) shown by the ^{1}H{^{11}B} NMR spectrum at 180 K are the same as those of the ^{1}H NMR spectrum at 180 K.

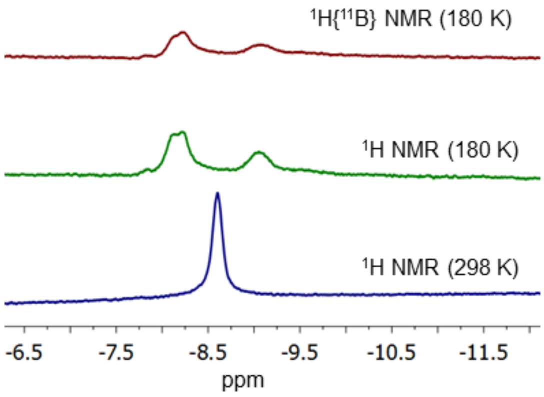

Figure S1 ^{1}H and ^{1}H{^{11}B} NMR spectra of **4** showing the hydride region.

^{31}P{^{1}H} NMR (202 MHz, d$_8$-THF): δ 40.8 (d, J_{RhP} = 142).

^{31}P{^{1}H} NMR (202 MHz, d$_8$-THF, 180 K): δ 41.6 (br d, J_{RhP} = 140), 36.1 (br d, J_{RhP} = 140).

^{11}B NMR (160 MHz, d$_8$-THF): δ 51.1 (br, BH$_2$), −6.5 (s, [BAr$^F_4$]$^-$).

ESI-MS (1,2-C$_6$H$_4$F$_2$, 60 °C) positive ion: m/z 788.2897 [M+] (calc. 788.2952).

Elemental Microanalysis: Calc. [C$_{62}$H$_{85}$B$_2$F$_{24}$NP$_4$Rh$_2$] (1651.6567gmol^{-1}): C, 45.09; H, 5.19; N, 0.85. Found: C, 44.42; H, 5.12; N, 0.94.

[Rh(PPh$_2$(CH$_2$)$_3$PPh$_2$)(THF)$_2$][BAr$^F_4$] (6)

THF (1 mL) was added to a Schlenk flask containing [Rh(PPh$_2$(CH$_2$)$_3$PPh$_2$)(C$_6$H$_5$F)][BAr$^F_4$] (50 mg, 3.4 x 10^{-2} mmol) and stirred for 15 minutes. Resulting orange solution was layered

with pentane at −18 ºC to yield [Rh($PPh_2(CH_2)_3PPh_2$)($THF)_2$][BAr^F_4] as orange crystals. Yield: 30 mg, 60%.

^{1}H NMR (500 MHz, d8-THF): δ 7.82 (s, 8H, [$BAr^F_4]^-$), 7.62 (s, 4H, [$BAr^F_4]^-$), 7.61 (br, 8 H, Ph) 7.47 (br, 12H, Ph), 2.50 (br, 8H, OCH_2), 1.35-0.93 (m, 8H, CH2), 0.15 (br, 6H, $CH_2CH_2CH_2$),

^{31}P{^{1}H} NMR (202 MHz, d8-THF): δ 37.3 (d, J_{RhP} = 192).

ESI-MS (THF, 60 °C) positive ion: m/z 659.1790 [M+] (calc. 659.1710).

Elemental Microanalysis: Calc. [$C_{50}H_{58}B_2F_{24}$NP2Rh] (1315.2897 $gmol^{-1}$): C, 52.85; H, 3.57. Found: C, 52.95; H, 3.10.

[Rh($P^iPr_2(CH_2)_3P^iPr_2$)(η^2-$H_3B\cdot NMe_3$)][BAr^F_4] (7)

[Rh($P^iPr_2(CH_2)_3P^iPr_2$)(C_6H_5F)][BAr^F_4] (32 mg, 2.4 x 10^{-2} mmol) and $H_3B\cdot NMe_3$ (1.8 mg, 2.4 x 10^{-2} mmol) were added to a Schlenk flask to which Et_2O (~1 mL) was added. Resulting solution was stirred for 2 h and then layered with pentane at −18 ºC to yield [Rh($P^iPr_2(CH_2)_3P^iPr_2$)(η^2-$H_3B\cdot NMe_3$)][BAr^F_4] as purple crystals. Yield: 20 mg, 64%.

^{1}H NMR (500 MHz, CD_2Cl_2): δ 7.76 (s, 8H, [$BAr^F_4]^-$), 7.60 (s, 4H, [$BAr^F_4]^-$), 2.89 (s, 9H, NMe_3), 1.94 (br, 6H, $CH_2CH_2CH_2$), 1.5 (br, 4H, CH), 1.23 (m, 24H, CH_3), −1.9 (br, 3H, BH_3).

^{31}P{^{1}H} NMR (202 MHz, CD_2Cl_2): δ 59.2 (d, J_{RhP} = 163).

^{11}B NMR (160 MHz, CD_2Cl_2): δ 24.5 (br, BH_2), −6.6 (s, [$BAr^F_4]^-$).

ESI-MS (1,2-$C_6H_4F_2$, 60 °C) positive ion: m/z 492.2548 [M+] (calc. 492.2565).

Elemental Microanalysis: Calc. [$C_{50}H_{58}B_2F_{24}NP_2$Rh] (1315.2897 $gmol^{-1}$): C, 45.65; H, 4.44; N, 1.06. Found: C, 45.95; H, 4.10; N, 1.12.

3. Variable temperature NMR spectra of 4 and Eyring analysis

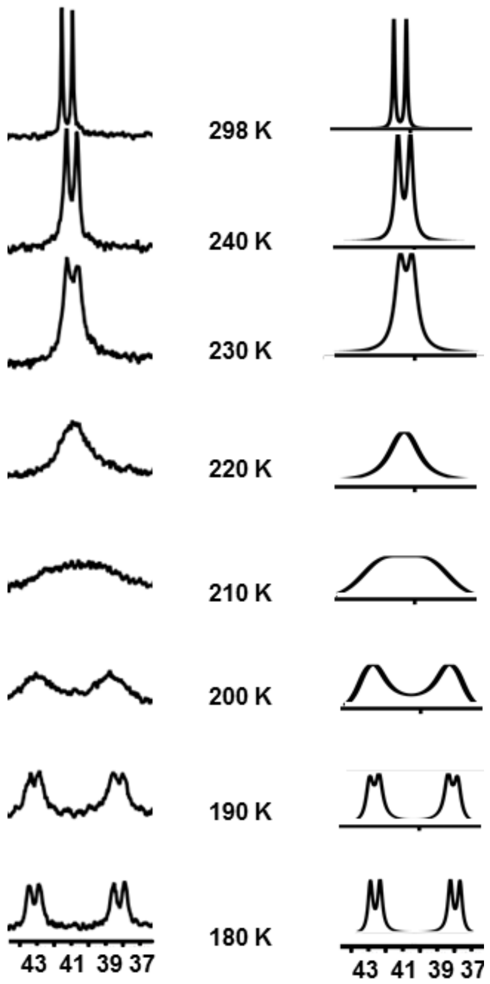

Figure S2 Representative $^{31}P\{^1H\}$ NMR spectra of $[(Rh(P^iPr_2(CH_2)_3P^iPr_2))_2(\mu\text{-}H)(\mu\text{-}BH_2NH_2)][BAr^F_4]$ (**4**) in THF at a range of temperatures (left) and their gNMR simulations (right).

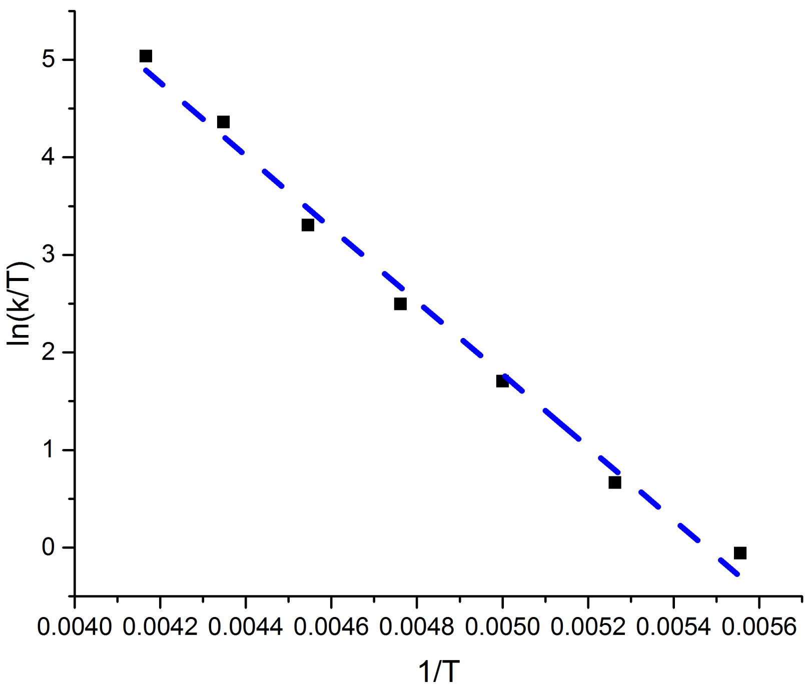

Figure S3 Eyring analysis for $[(Rh(P^iPr_2(CH_2)_3P^iPr_2))_2(\mu\text{-}H)(\mu\text{-}BH_2NH_2)][BAr^F_4]$. ln (k/T) = −3736.85(1/T) + 20.46

Activation parameters derived from the Eyring plot for the fluxional nature of amino-borane:

$\Delta H^\ddagger = 31.1 \pm 1.3$ kJ mol^{-1}, $\Delta S^\ddagger = -27.0 \pm 1.0$ J K^{-1} mol^{-1}, $\Delta G^\ddagger = 39.2 \pm 1.6$ kJ mol^{-1}.

4. Stoichiometric reactions of $H_3B{\cdot}NH_3$ with 1 and 2 in THF

Reaction with $[Rh(PPh_2(CH_2)_3PPh_2)(C_6H_5F)][BAr^F_4]$ (1)

1.2 equivalents of $H_3B{\cdot}NH_3$ was added to $[Rh(PPh_2(CH_2)_3PPh_2)(C_6H_5F)][BAr^F_4]$ (**1**, 20 mg, 1.4 x 10^{-2} mmol) in d-8 THF in a sealed NMR tube equipped with a J. Young's valve. NMR spectroscopy and ESI-MS after 2 minutes showed the formation of $[(Rh(PPh_2(CH_2)_3PPh_2))_2(\mu\text{-}H)(\mu\text{-}BH_2NH_2)][BAr^F_4]$ (**3**) as the only organometallic species. ^{11}B NMR spectrum also showed the formation of $[BH_2(NH_3)(THF)][BAr^F_4]$ (^{11}B NMR: δ 0.5, t, J_{BH} = 108). $[(Rh(PPh_2(CH_2)_3PPh_2))_2(\mu\text{-}H)(\mu\text{-}BH_2NH_2)][BAr^F_4]$ showed decomposition over time and after 4 h only 12% of $[(Rh(PPh_2(CH_2)_3PPh_2))_2(\mu\text{-}H)(\mu\text{-}BH_2NH_2)][BAr^F_4]$ was left, 30% of $[Rh(PPh_2(CH_2)_3PPh_2)(THF)_2][BAr^F_4]$ was also observed, rest being the uncharacterized complexes. Layering the THF solution (after 2 minutes of the reaction) with pentane at −18 ^{o}C resulted in a small number of crystals which were sufficient for the single crystal X-ray diffraction but not for the reliable NMR data or elemental microanalysis.

Spectral details after 2 minutes of reaction:

^{1}H NMR (500 MHz, d_8-THF): δ 7.79 (s, $[BAr^F_4]^-$), 7.57 (s, $[BAr^F_4]^-$), 7.49 – 7.24 (overlapping m, Ph), 5.67 (br, NH_2), 1.99 (br, CH_2), −7.45 (s, BH_2 and RhH). Signal at δ −7.45 showed integral of 3H relative to the phenyl resonance.

$^{31}P\{^1H\}$ NMR (202 MHz, d_8-THF): δ 18.2 (d, J_{RhP} = 142).

^{11}B NMR (160 MHz, d_8-THF): δ 51.5 (br, BH_2), −6.5 (s, $[BAr^F_4]^-$), 0.5 (t, J_{BH} = 108, corresponding to $[BH_2(NH_3)(THF)][BAr^F_4]$).

ESI-MS (1,2-$C_6H_4F_2$, 60 °C) positive ion: m/z 1060.1612 [M+] (calc. 1060.1645).

Reactions with $[Rh(P^iPr_2(CH_2)_3P^iPr_2)(C_6H_5F)][BAr^F_4]$ (2)

THF (0.35 mL) was added to a sealed NMR tube equipped with a J. Young's valve and charged with $[Rh(P^iPr_2(CH_2)_3P^iPr_2)(C_6H_5F)][BAr^F_4]$ (16 mg, 1.2 x 10^{-2} mmol) and $H_3B{\cdot}NH_3$ (~0.5 mg, 1.4 x 10^{-2} mmol). NMR spectroscopy after 2 minutes showed the formation of $[Rh(P^iPr_2(CH_2)_3P^iPr_2)(\eta^2\text{-}H_3B{\cdot}NH_3)][BAr^F_4]$ and $[(Rh(P^iPr_2(CH_2)_3P^iPr_2))_2(\mu\text{-}H)(\mu\text{-}BH_2NH_2)][BAr^F_4]$ in a 7:3 ratio along with the formation of $[BH_2(NH_3)(THF)][BAr^F_4]$ (^{11}B NMR: δ 0.5, t, J_{BH} = 108). Over time the concentration of the two complexes changes leading to a relative increment in the formation of $[(Rh(P^iPr_2(CH_2)_3P^iPr_2))_2(\mu\text{-}H)(\mu\text{-}BH_2NH_2)][BAr^F_4]$. NMR spectroscopy after 1 h showed the presence of $[Rh(P^iPr_2(CH_2)_3P^iPr_2)(\eta^2\text{-}H_3B{\cdot}NH_3)][BAr^F_4]$ and $[(Rh(P^iPr_2(CH_2)_3P^iPr_2))_2(\mu\text{-}H)(\mu\text{-}BH_2NH_2)][BAr^F_4]$ in a 6:4 ratio.

Characteristic NMR signals corresponding to $[Rh(P^iPr_2(CH_2)_3P^iPr_2)(\eta^2\text{-}H_3B{\cdot}NH_3)][BAr^F_4]$

^{1}H NMR (500 MHz, THF): δ −1.65 (br, bound BH_2).

$^{31}P\{^1H\}$ NMR (202 MHz, THF): δ 59.0 (d, J_{RhP} = 160).

^{11}B NMR (160 MHz, THF): δ 17.0 (br, BH_2), -6.5 (s, $[BAr^F_4]^-$), 0.5 (t, J_{BH} = 108, corresponding to $[BH_2(NH_3)(THF)][BAr^F_4]$).

5. Reaction of 4 equivalents of $H_3B{\cdot}NH_3$ with 2 in THF

THF (0.35 mL) was added to a sealed NMR tube charged with $[Rh(P^iPr_2(CH_2)_3P^iPr_2)(C_6H_5F)][BAr^F_4]$ (8 mg, 0.6 x 10^{-2} mmol) and $H_3B{\cdot}NH_3$ (~0.7 mg, 2.4 x 10^{-2} mmol). NMR spectroscopy after 2 minutes showed the formation of $[Rh(P^iPr_2(CH_2)_3P^iPr_2)(\eta^2\text{-}H_3B{\cdot}NH_3)][BAr^F_4]$ and $[(Rh(P^iPr_2(CH_2)_3P^iPr_2))_2(\mu\text{-}H)(\mu\text{-}BH_2NH_2)][BAr^F_4]$ in 1:3 ratio along with the formation of $[BH_2(NH_3)(THF)][BAr^F_4]$ (^{11}B NMR: δ 0.5, t, J_{BH}

= 108). At this stage NMR signals due to free $H_3B{\cdot}NH_3$ are broad in ^{11}B NMR spectrum thus suggesting a rapid exchange between the bound and the free $H_3B{\cdot}NH_3$. After 10 minutes all $[Rh(P^iPr_2(CH_2)_3P^iPr_2)(\eta^2\text{-}H_3B{\cdot}NH_3)][BAr^F{}_4]$ and $[BH_2(NH_3)(THF)][BAr^F{}_4]$ gets consumed and $[(Rh(P^iPr_2(CH_2)_3P^iPr_2))_2(\mu\text{-}H)(\mu\text{-}BH_2NH_2)][BAr^F{}_4]$ was found to be the only organometallic species detected by $^{31}P\{^1H\}$ NMR spectroscopy. As the consumption of $[Rh(P^iPr_2(CH_2)_3P^iPr_2)(\eta^2\text{-}H_3B{\cdot}NH_3)][BAr^F{}_4]$ reduced the opportunity of exchange between free and bound $H_3B{\cdot}NH_3$, free $H_3B{\cdot}NH_3$ was observed as a quartet in ^{11}B NMR spectrum (δ −21.9, q, J_{BH} = 100). After 24 h $[(Rh(P^iPr_2(CH_2)_3P^iPr_2))_2(\mu\text{-}H)(\mu\text{-}BH_2NH_2)][BAr^F{}_4]$ got almost completely consumed with the formation of $[\{Rh(P^iPr_2(CH_2)_3P^iPr_2)\}_2(H)_2(\mu\text{-}H)_3][BAr^F{}_4]$[8] and the same uncharacterized species ($^{31}P\{^1H\}$ NMR: δ 56.0 and 51.0, JRhP = ~111 Hz) in 1:1 ratio, ratio remaining the same for the next 3 days.

6. General method for the catalytic dehydrocoupling (open system)

Rhodium catalyst (e.g. **1**, 8 mg, 5.4 x 10^{-3} mmol) and amine-borane (e.g. $H_3B{\cdot}NH_2Me$, 48 mg, 1.06 mmol, ~200 equivalents) were added to a 3-necked Schlenk flask which was connected to an external mineral oil bubbler. Addiiton of solvent (1,2-$F_2C_6H_4$ or THF, 2.6 mL) started the dehydrogenation experiment. Evolution of hydrogen gas vs. time was monitored by attaching a cannula to the Schlenk, the other end of cannula was placed under an upturned 50 mL burette filled with water.

7. Catalytic dehydrocoupling of $H_3B{\cdot}NH_3$ using 0.5 mol% 1 in THF

The reaction of $[Rh(PPh_2(CH_2)_3PPh_2)(C_6H_5F)][BAr^F{}_4]$ (**1**, 2 x 10^{-3} M) and $H_3B{\cdot}NH_3$ (0.41 M) in THF as described in Section 6 immediately produced bubbles indicating the immediate start of the dehydrogenation process. $H_3B{\cdot}NH_3$ was completely consumed in 3 h producing 1.2 equivalents of H_2 gas and some insoluble solid. ^{11}B NMR spectrum of the reaction mixture after 3 h showed the formation of borazine and polyborazylene along with the three unresolved broad signals at δ −5.3, −12.8 and −24.6 which could possibly be due to the mixture of B-(cyclotriborazanyl)amine-borane (BCTB)[9] and short chain B−N oligomers. Insoluble solid was separated by filtration whose IR spectrum is suggestive of polyaminoborane reported by Manners and co-workers.[10] THF solution was then concentrated to which toluene was added to precipitate out the product. Product was purified by washing with toluene. The isolated product was characterized as the mixture of B-(cyclotriborazanyl)amine-borane (BCTB) and the short chain B−N oligomers[11] through the ^{11}B NMR spectroscopy.

Hydrogen evolution experiment:

95% conversion in 3h (TOF ~ 60 h^{-1}): release of 1.25 equivalents of H_2.

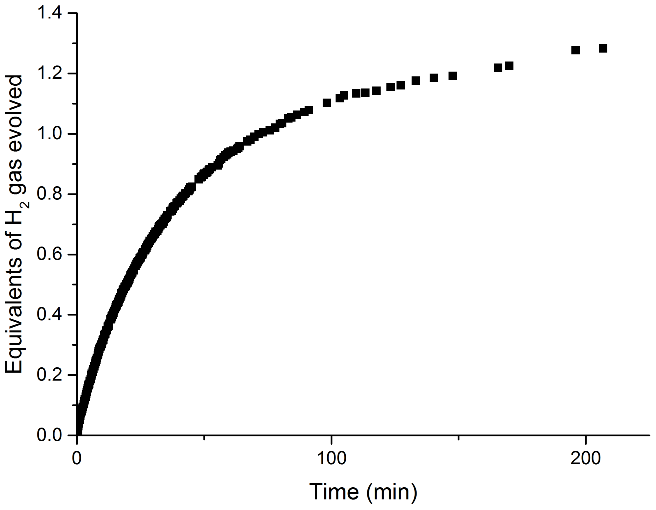

Figure S4 Plot of equivalents of H_2 gas produced vs time during dehydrocoupling of $H_3B{\cdot}NH_3$ (0.41 M) using $[Rh(PPh_2(CH_2)_3PPh_2)(C_6H_5F)][BAr^F{}_4]$ (**1**, 2 x 10^{-3} M, 0.5 mol%).

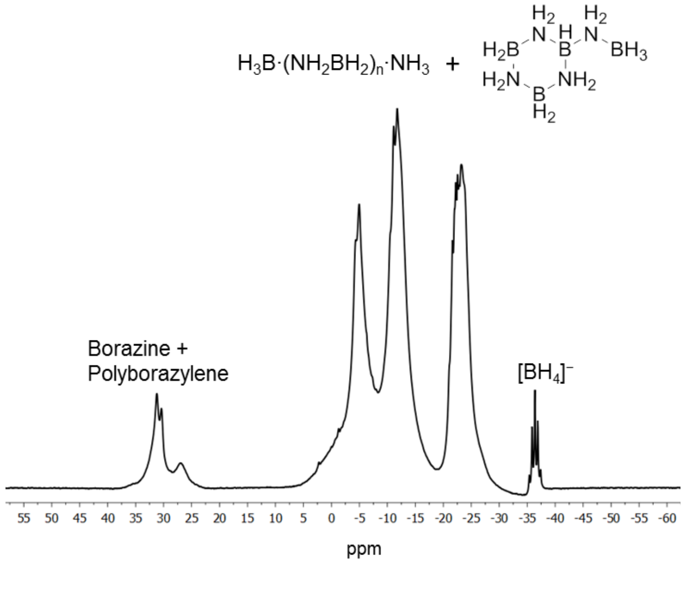

Figure S5 ^{11}B NMR spectrum of the reaction mixture after 3 h of dehydrocoupling of $H_3B{\cdot}NH_3$ (0.41 M) using $[Rh(PPh_2(CH_2)_3PPh_2)(C_6H_5F)][BAr^F{}_4]$ (**1**, 2 x 10^{-3} M, 0.5 mol%).

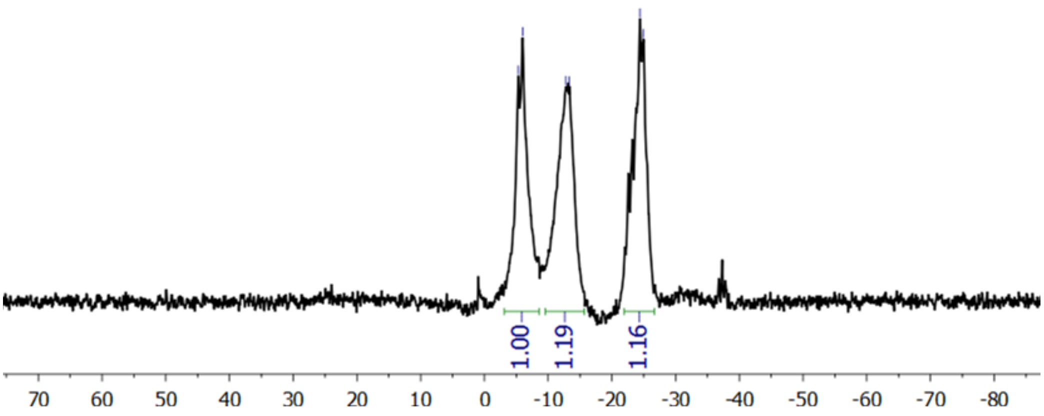

Figure S6 ^{11}B NMR spectrum of the isolated product from the catalytic dehydrocoupling of $H_3B{\cdot}NH_3$ (0. 41 M) using [Rh(PPh$_2$(CH$_2$)$_3$PPh$_2$)(C$_6$H$_5$F)][BAr$^F{}_4$] (**1**, 2 x 10^{-3} M, 0.5 mol %).

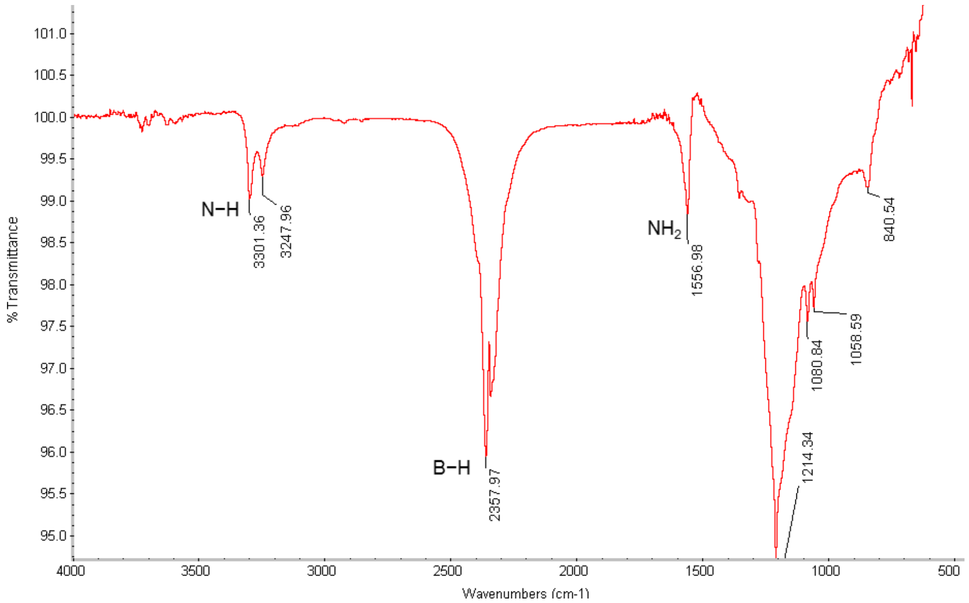

Figure S7 IR spectrum of white insoluble product obtained from the dehydrocoupling of $H_3B{\cdot}NH_3$ (0. 41 M) using [Rh(PPh$_2$(CH$_2$)$_3$PPh$_2$)(C$_6$H$_5$F)][BAr$^F{}_4$] (**1**, 2 x 10^{-3} M, 0.5 mol%).

8. Catalytic dehydrogenation of $H_3B{\cdot}NH_3$ using 0.5 mol% [BH$_2$(NH$_3$)(OEt$_2$)][BAr$^F{}_4$] in THF

No dehydrogenation was observed on reaction of $H_3B{\cdot}NH_3$ (0.41 M) with [BH$_2$(NH$_3$)(OEt$_2$)][BAr$^F{}_4$] (2 x 10^{-3} M, 0.5 mol%) as monitored by the gas-burette method for 3 h. ^{11}B NMR spectrum after 3 h showed only the signals corresponding to $H_3B{\cdot}NH_3$ (δ −21.9, q, J_{BH} = 100).

9. Catalytic dehydrocoupling of $H_3B{\cdot}NH_3$ using 2 (4 mol%) or $[\{Rh(P^iPr_2(CH_2)_3P^iPr_2)\}_2(H)_2(\mu\text{-}H)_3][BAr^F_4]$ (2 mol%) in THF in a closed system

The catalytic dehydrocoupling of $H_3B{\cdot}NH_3$ was conducted using $[Rh(P^iPr_2(CH_2)_3P^iPr_2)(C_6H_5F)][BAr^F_4]$ (**2**) (4 mol%) or $[\{Rh(P^iPr_2(CH_2)_3P^iPr_2)\}_2(H)_2(\mu\text{-}H)_3][BAr^F_4]$ (2 mol%) in THF in a sealed NMR tube. Complex **2** gave ~50% conversion while $[\{Rh(P^iPr_2(CH_2)_3P^iPr_2)\}_2(H)_2(\mu\text{-}H)_3][BAr^F_4]$ resulted in ~30% conversion of $H_3B{\cdot}NH_3$ in 10 h (Figure S8). ^{11}B NMR spectroscopy showed the formation of a mixture of species containing borazine, B-(cyclotriborazanyl)amine-borane[9] and short chain B−N oligomers. A broad unidentified resonance at δ −43 was also observed in the ^{11}B NMR spectrum at the end of both dehydrocoupling experiments.

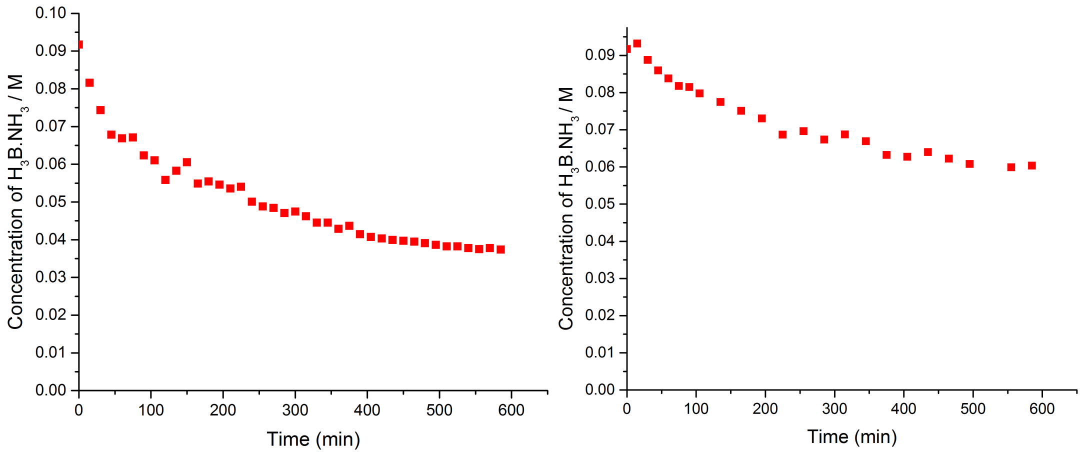

Figure S8 Comparison plots of concentration of $H_3B{\cdot}NH_3$ against time for the catalytic dehydrocoupling of $H_3B{\cdot}NH_3$ (9×10^{-2} M) in THF (sealed system). Left: complex **2** (3.6×10^{-3} M, 4 mol%); Right: $[\{Rh(P^iPr_2(CH_2)_3P^iPr_2)\}_2(H)_2(\mu\text{-}H)_3][BAr^F_4]$ (1.7×10^{-3} M, 2 mol%).

10. Catalytic dehydrocoupling of $H_3B{\cdot}NH_3$ using 2 (4 mol%) or 4 (2 mol%) in THF in a closed system

$H_3B{\cdot}NH_3$ (9×10^{-2} M in THF) and the appropriate rhodium complex (**2**, 4 mol% or **4**, 2 mol%) were added to a sealed NMR tube and the reaction progress was monitored using ^{11}B NMR spectroscopy. Catalyst **2** resulted in ~50% conversion of $H_3B{\cdot}NH_3$ to the mixture of dehydrocoupling products containing polyaminoborane, borazine, B-(cyclotriborazanyl)amine-borane (BCTB)[9] and polyborazylene in 10 h whereas **4** resulted in 70% conversion of $H_3B{\cdot}NH_3$ in 10 h, yielding the same dehydrocoupling products as obtained with **2**.

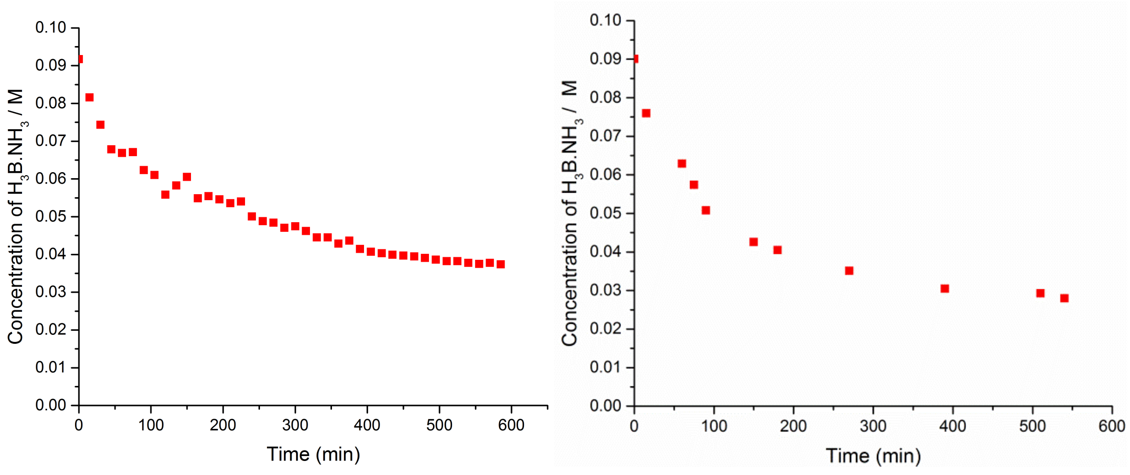

Figure S9 Comparison plots of concentration of $H_3B{\cdot}NH_3$ against time for the catalytic dehydrocoupling of $H_3B{\cdot}NH_3$ (initial concentration: 9 x 10^{-2} M) in THF (sealed system). Left: using complex **2** (3.6 x 10^{-3} M, 4 mol %; see Fig. S8); Right: using complex **4** (1.7 x 10^{-3} M, 2 mol%).

We suggest that the drop off in catalytic activity of **2** and **4** is due to the catalyst deactivation by the presence of excess H_2 leading to the formation of a less active catalyst [{Rh(P^{i}Pr$_2$(CH$_2$)$_3$P^{i}Pr$_2$)}$_2$(H)$_2$(μ-H)$_3$][BAr$^F{}_4$], which has been shown to be a final product of reaction with excess $H_3B{\cdot}NH_3$ on a sealed system and also to form on direct addition of H_2 to **4**. To probe the role of H_2 the dehydrocoupling of $H_3B{\cdot}NH_3$ was monitored using ^{11}B NMR spectroscopy in a sealed system under the same catalytic condition: $H_3B{\cdot}NH_3$, 9 x 10^{-2} M; **2**, 4 mol%; THF. Almost 50% conversion of $H_3B{\cdot}NH_3$ was obtained in 3.5 h after which the sample was degassed using freeze-pump-thaw method. Further monitoring the reaction using ^{11}B NMR spectroscopy revealed that the rate of dehydrogenation after degassing is faster than the rate just before degassing (Figure S10). We speculate this is because on degassing, the deactivated catalyst [{Rh(P^{i}Pr$_2$(CH$_2$)$_3$P^{i}Pr$_2$)}$_2$(H)$_2$(μ-H)$_3$][BAr$^F{}_4$] reforms [{Rh(P^{i}Pr$_2$(CH$_2$)$_3$P^{i}Pr$_2$)}$_2$(μ-H)(μ-BH$_2$NH$_2$)][BAr$^F{}_4$] (**4**) which shows greater catalytic activity. Consistent with the regeneration of a more active catalyst in the absence of H_2, addition of two equivalents of $H_3B{\cdot}NH_3$ to pure [{Rh(P^{i}Pr$_2$(CH$_2$)$_3$P^{i}Pr$_2$)}$_2$(H)$_2$(μ-H)$_3$][BAr$^F{}_4$] resulted in the immediate formation of [(Rh(P^{i}Pr$_2$(CH$_2$)$_3$P^{i}Pr$_2$))$_2$(μ-H)(μ-BH$_2$NH$_2$)][BAr$^F{}_4$] (**4**, 42%) and some uncharacterized organometallic complexes (12%), with the rest being the starting complex [{Rh(P^{i}Pr$_2$(CH$_2$)$_3$P^{i}Pr$_2$)}$_2$(H)$_2$(μ-H)$_3$][BAr$^F{}_4$] (46%), as observed by the ^{31}P{^{1}H} NMR spectroscopy. The $H_3B{\cdot}NH_3$ was completely converted to borazine and polyaminoborane in 24 h. The ^{31}P{^{1}H} NMR spectrum at this point showed the presence of **4** (23%), [{Rh(P^{i}Pr$_2$(CH$_2$)$_3$P^{i}Pr$_2$)}$_2$(H)$_2$(μ-H)$_3$][BAr$^F{}_4$] (43%) and some uncharacterized organometallic complexes (34%). This experiment, combined with the reaction of **4** with hydrogen to form [{Rh(P^{i}Pr$_2$(CH$_2$)$_3$P^{i}Pr$_2$)}$_2$(H)$_2$(μ-H)$_3$][BAr$^F{}_4$], suggests an equilibrium is established between

[{Rh(P^{i}Pr$_2$(CH$_2$)$_3$P^{i}Pr$_2$)}$_2$(H)$_2$(μ-H)$_3$][BAr$^F_4$] and **4** that is biased, respectively, by H_2 and $H_3B{\cdot}NH_3$.

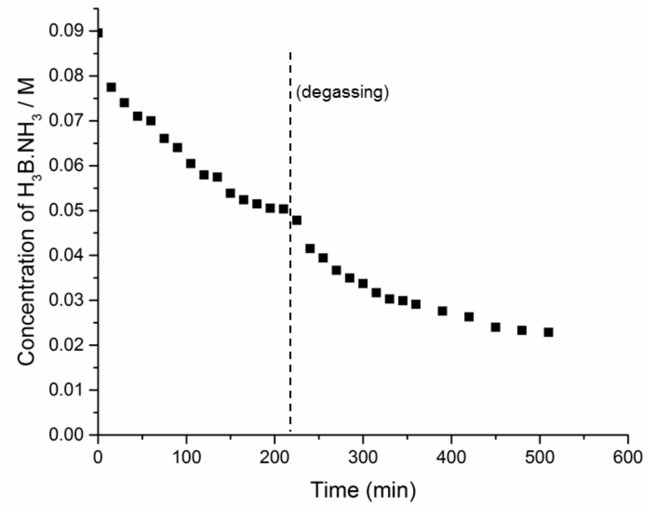

Figure S10 Plot of concentration of $H_3B{\cdot}NH_3$ against time for the dehydrocoupling of $H_3B{\cdot}NH_3$ (9 x 10^{-2} M) using catalyst **2** (3.6 x 10^{-3} M, 4 mol%) in THF (sealed system). After 3.5 h, the system was opened to an Ar atmosphere, feeeze-pump-thaw degassed and closed.

11. Reactivity of $H_3B{\cdot}NH_2Me$ with 1 in THF: Formation of 8

1 equivalent of $H_3B{\cdot}NH_2Me$ was added to [Rh(PPh$_2$(CH$_2$)$_3$PPh$_2$)(C$_6$H$_5$F)][BAr$^F_4$] (**1**) in THF in a sealed NMR tube. NMR spectroscopy and ESI-MS (m/z 1074.1340 [M+], calc. 1074.1801) after 2 minutes showed the characteristic signals corresponding to the formation of [(Rh(PPh$_2$(CH$_2$)$_3$PPh$_2$))$_2$(μ-H)(μ-BH$_2$NHMe)][BAr$^F_4$] (**8**) as the only organometallic compound. ^{1}H NMR showed signal at δ −6.84 (s, 3H, relative to the phenyl groups) corresponding to bound hydrides (BH$_2$ and RhH). ^{31}P{^{1}H} NMR spectroscopy showed two broad signals at δ 22.2 and 21.5 both integrating one relative to each other. Bound H$_2$B=NHMe unit was observed at δ 50.6 in ^{11}B NMR spectrum. Signal corresponding to [BH$_2$(NH$_2$Me)(THF)][BAr$^F_4$] was also observed in ^{11}B NMR spectrum at δ 2.8 (t, J_{BH} = 123) which is consistent with [BH$_2$(NH$_2$Me)(OEt$_2$)][B(C$_6$F$_5$)$_4$] reported by Manners and co-workers [CD$_2$Cl$_2$, δ 1.7 (t, J_{BH} = 121)].[12]

12. Reactivity of $H_3B{\cdot}NH_2Me$ with 1 in 1,2-$F_2C_6H_4$: Formation of 8

With 1.2 eq of $H_3B{\cdot}NH_2Me$

1.2 equivalents of $H_3B{\cdot}NH_2Me$ was added to [Rh(PPh$_2$(CH$_2$)$_3$PPh$_2$)(C$_6$H$_5$F)][BAr$^F_4$] (**1**) in 1,2-F$_2$C$_6$H$_4$ in a sealed NMR tube. NMR spectroscopy after 2 minutes showed the formation of the dimer, [(Rh(PPh$_2$(CH$_2$)$_3$PPh$_2$))$_2$(μ-H)(μ-BH$_2$NHMe)][BAr$^F_4$] (**8**) in 5% yield, the rest being the starting complex **1**. ^{11}B NMR spectrum showed signal at δ −7.7 (t, J_{BH} = 115), suggestive

of the formation of $[(NH_2Me)_2BH_2][BAr^F{}_4]$.[13,14] Formation of the bridged species $H_2B(\mu\text{-}H)(NMeH)BH_2$ [δ −22.0, td, J_{HB} = 32 and 130][15] was also observed through ^{11}B NMR spectroscopy. Formation of $[(NH_2Me)_2BH_2][BAr^F{}_4]$ could result from the reaction of $[BH_2(NH_2Me)(S)][BAr^F{}_4]$ (S = solvent)* with NH_2Me resulting from the B−N cleavage of $H_3B{\cdot}NH_2Me$ as previously reported during Pt (II) catalysed dehydrocoupling of $H_3B{\cdot}NMe_2H$.[14] BH_3, can then react with the amino-borane $H_2B{=}NHMe$ or $H_3B{\cdot}NH_2Me$ (release H_2) to form $(BH_2)_2NMeH(\mu\text{-}H)$. After 24 h, $^{31}P\{^1H\}$ NMR spectra shows complex **1** as the only organometallic compound present in the reaction mixture. ^{11}B NMR spectroscopy shows the formation of borazine and $[(NH_2Me)_2BH_2][BAr^F{}_4]$.

*Formation of $[BH_2(NH_2Me)(S)][BAr^F{}_4]$ is observed as an intermediate during the formation of **8** in nucleophilic solvents, for example THF or Et_2O (Scheme 5).

With 4 eq of $H_3B{\cdot}NH_2Me$

4 equivalents of $H_3B{\cdot}NH_2Me$ was added to $[Rh(PPh_2(CH_2)_3PPh_2)(C_6H_5F)][BAr^F{}_4]$ (**1**) in 1,2-$F_2C_6H_4$ in a sealed NMR tube. NMR spectroscopy after 2 minutes showed the formation of the dimer, $[(Rh(PPh_2(CH_2)_3PPh_2))_2(\mu\text{-}H)(\mu\text{-}BH_2NHMe)][BAr^F{}_4]$ (**8**) in 90% yield and some uncharacterized complex (~10% yield). Formation of $[Rh(PPh_2(CH_2)_3PPh_2)(\eta^2\text{-}H_3B{\cdot}NH_2Me)][BAr^F{}_4]$ was not observed. ^{11}B NMR spectroscopy showed the formation of the cyclic product $(BH_2)_2NMeH(\mu\text{-}H)$ and polymethylaminoborane (δ br, −5.5). Signal due to $[(NH_2Me)_2BH_2][BAr^F{}_4]$ in the ^{11}B NMR spectrum is obscured by the broad signal of the polymer. NMR spectra after 20 minutes showed complete decomposition of the dimer (**8**) to give some uncharacterized complexes.

13. Catalytic dehydrocoupling of $H_3B{\cdot}NH_2Me$ using 0.5 mol% 1 in THF

1.15 equivalents of H_2 gas was released as measured by the gas burette method as described in general experimental procedure (Section 6).

Formation of polyaminoborane ($[H_2BNMeH]_n$, ~95%) and borazine ($[HBNMe]_3$, ~5%) was observed through ^{11}B NMR spectroscopy.

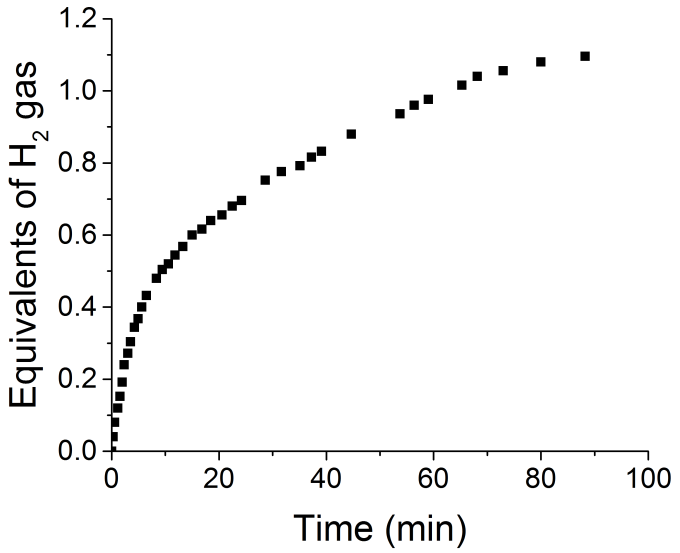

Figure S11 Plot of equivalents of H_2 gas produced vs time during the dehydrocoupling of $H_3B{\cdot}NH_2Me$ (0. 41 M) using $[Rh(PPh_2(CH_2)_3PPh_2)(C_6H_5F)][BAr^F{}_4]$ (**1**, 2 x 10^{-3} M, 0.5 mol%) in THF.

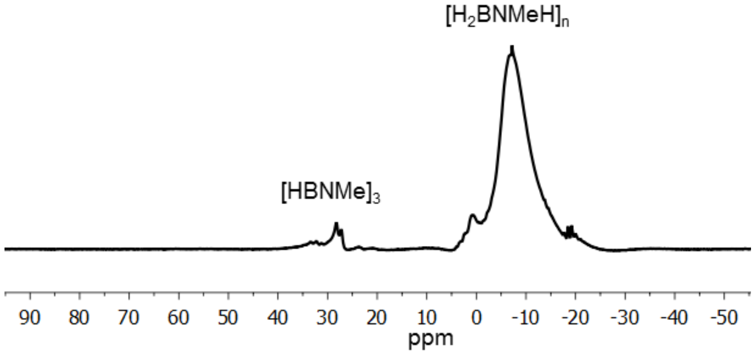

Figure S12 ^{11}B NMR spectrum at the end of the catalytic dehydrocoupling of $H_3B{\cdot}NH_2Me$ (0. 41 M) using $[Rh(PPh_2(CH_2)_3PPh_2)(C_6H_5F)][BAr^F{}_4]$ (**1**, 2 x 10^{-3} M, 0.5 mol%) in THF.

Polymer from the catalytic dehydrocoupling of $H_3B{\cdot}NH_2Me$ (0.41 M) using **III** (2 x 10^{-3} M, 0.5 mol%, THF) was isolated as per the literature procedure[5] by precipitating it in the hexane solvent and then subsequent filtration, washing with hexane and drying steps. Yield: 18 mg, 40%.

GPC data

M_w = 30,600; M_n = 11,740; $Đ$ = 2.6.

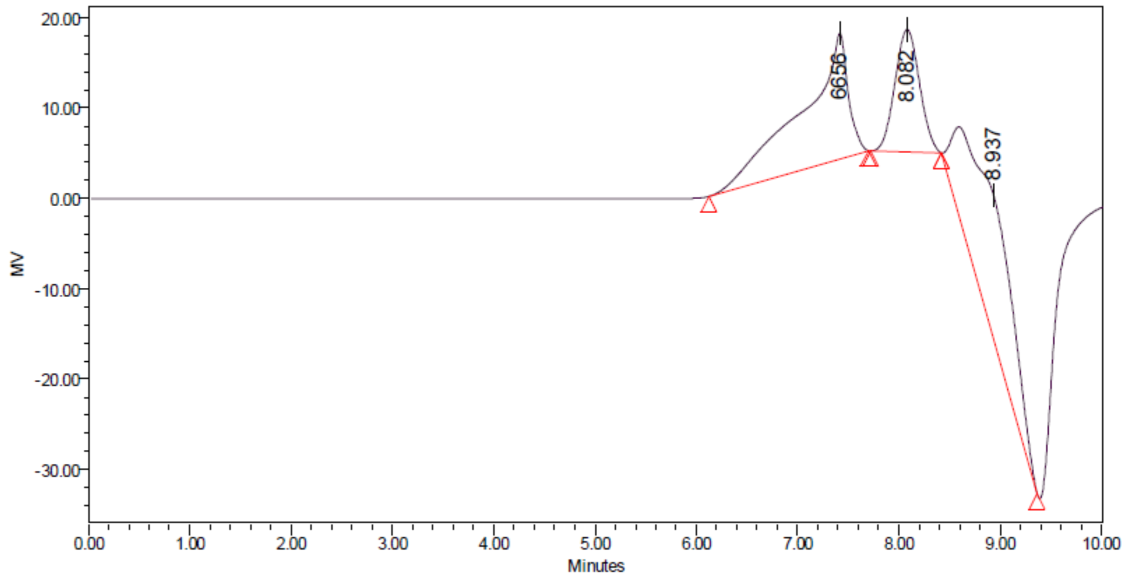

Figure S13 Gel permeation chromatogram (plot of refractive index response against time) for the isolated polymer. The peak at t = 8.082 is a system peak.

14. Catalytic dehydrocoupling of $H_3B{\cdot}NH_2Me$ using 0.5 mol% 1 in 1,2-$F_2C_6H_4$

1.1 equivalents of H_2 gas was released as measured by the gas burette method as described in general experimental procedure (Section 6).

Formation of polyaminoborane ($[H_2BNMeH]_n$, ~95 %) and borazine ($[HBNMe]_3$, ~5 %) was observed through the ^{11}B NMR spectroscopy.

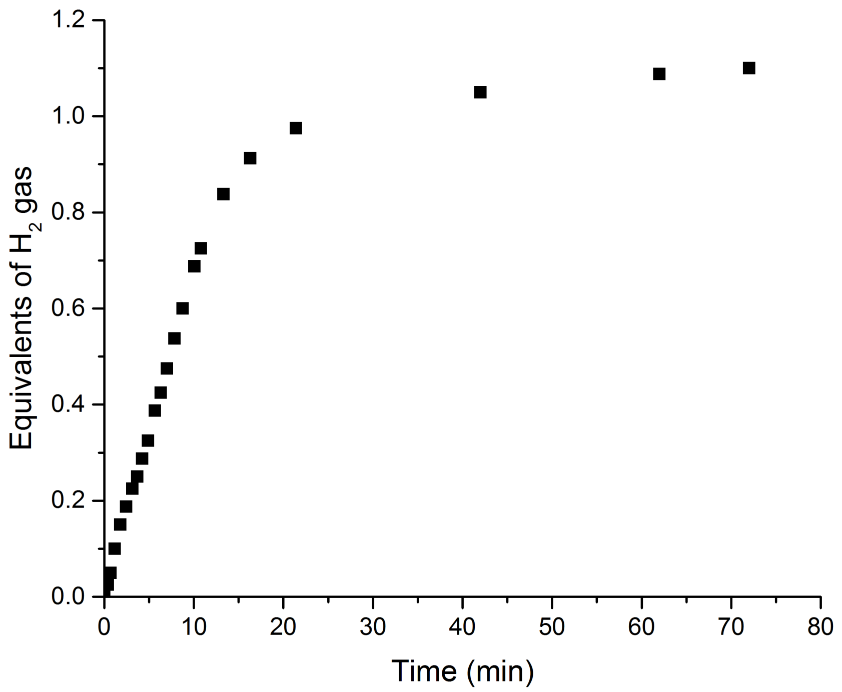

Figure S14 Plot of equivalents of H_2 gas produced vs time during dehydrocoupling of $H_3B{\cdot}NH_2Me$ (0. 41 M) using $[Rh(PPh_2(CH_2)_3PPh_2)(C_6H_5F)][BAr^F{}_4]$ (**1**, 2 x 10^{-3} M, 0.5 mol %) in 1,2-$F_2C_6H_4$.

15. Preparation (*in situ*) of 8 in THF

1 equivalent of $H_3B{\cdot}NH_2Me$ (1 mg, 0.022 mmol) was added to $[Rh(PPh_2(CH_2)_3PPh_2)(C_6H_5F)][BAr^F{}_4]$ (**1,** 32 mg, 0.022 mmol) in THF (0.5 mL) in a Schlenk flask. Reaction mixture was stirred for two minutes, evacuated, washed with pentane and then dried to obtain orange powder of **8**. $[BH_2(NH_2Me)(THF)][BAr^F{}_4]$ was also present in the obtained solid. The orange solid was then used as catalyst for the $H_3B{\cdot}NH_2Me$ dehydrocoupling experiments.

16. Catalytic dehydrocoupling of $H_3B{\cdot}NH_2Me$ using *in situ* prepared 8 (0.25 mol%) in THF

1.13 equivalents of H_2 gas was released as measured by gas burette method as described in general experimental procedure (Section 6).

Formation of polyaminoborane ($[H_2BNMeH]_n$, ~95%) and borazine ($[HBNMe]_3$, ~5%) was observed through ^{11}B NMR spectroscopy.

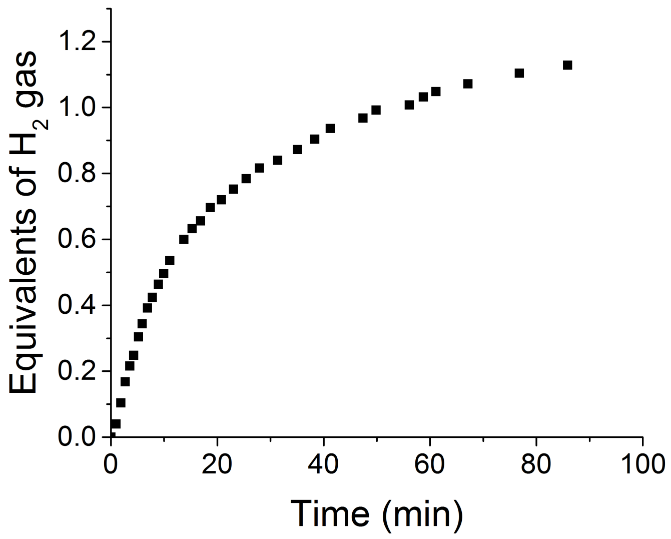

Figure S15 Plot of equivalents of H_2 gas produced vs time during dehydrocoupling of $H_3B{\cdot}NH_2Me$ (0. 41 M) using *in situ* prepared **8** (1 x 10^{-3} M, 0.25 mol%) in THF.

17. Catalytic dehydrocoupling of $H_3B{\cdot}NH_2Me$ using *in situ* prepared 8 (0.25 mol%) in 1,2-$F_2C_6H_4$

1.12 equivalents of H_2 gas was released as measured by the gas burette method as described in general experimental procedure (Section 6).

Formation of polyaminoborane ($[H_2BNMeH]_n$, ~95%) and borazine ($[HBNMe]_3$, ~5%) was observed through the ^{11}B NMR spectroscopy.

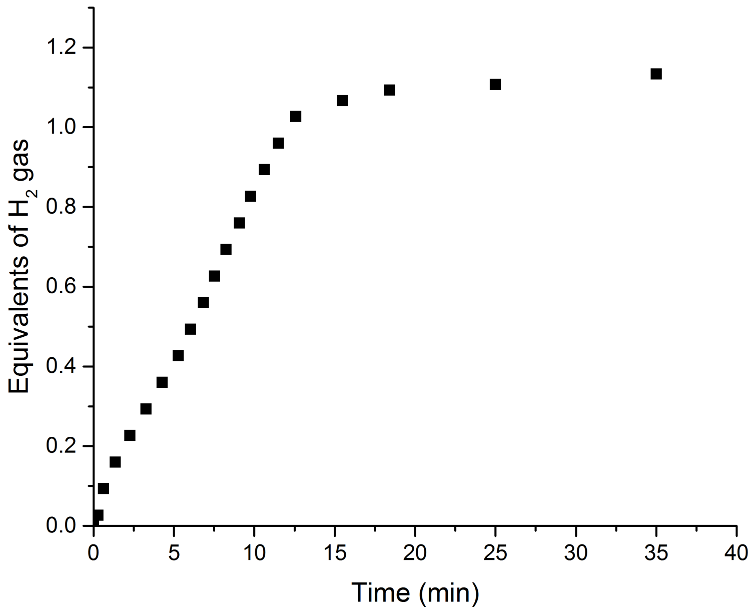

Figure S16 Plot of equivalents of H_2 gas produced vs time during dehydrocoupling of $H_3B{\cdot}NH_2Me$ (0. 41 M) using *in situ* prepared **8** (1 x 10^{-3} M, 0.25 mol%) in 1,2-$F_2C_6H_4$.

18. Catalytic dehydrocoupling of $H_3B{\cdot}NMe_2H$ in 1,2-$F_2C_6H_4$

0.7 mL solution of **1** (2×10^{-3} M, 0.5 mol %) in 1,2-$F_2C_6H_4$ was added to $H_3B{\cdot}NMe_2H$ (0. 41 M) and the evolution of hydrogen gas was measured using gas-burette method which showed the release of one equivalent of H_2 gas in 30 minutes. The temporal plot of evolution of hydrogen gas (Figure S17) shows a sigmoidal profile with a slow release of 0.3 equivalent of H_2 gas in the first 10 minutes followed by a highly rapid release of 0.6 equivalent of H_2 gas in the next 2.5 minutes and then a very slow evolution of 0.1 equivalent of H_2 gas in the next 18 minutes. ^{11}B NMR spectroscopy at the end of the reaction (30 minutes) showed the complete conversion of $H_3B{\cdot}NMe_2H$ to $[H_2BNMe_2]_2$. The slow release of hydrogen gas at the start (10 minutes) can be attributed to the induction period. We suggest that the induction period is related to the slow formation of the dimer $[\{Rh(PPh_2(CH_2)_3PPh_2)\}_2(\mu\text{-}H)(\mu\text{-}BH_2NMe_2)][BAr^F_4]$ from **1**, but cannot discount that $[\{Rh(PPh_2(CH_2)_3PPh_2)\}_2(\mu\text{-}H)(\mu\text{-}BH_2NMe_2)][BAr^F_4]$ is a precatalyst. The observation of induction period (measured by ^{11}B NMR spectroscopy) as well as the homogeneous nature of the catalysis for this reaction (i.e. the dehydrocoupling of $H_3B{\cdot}NMe_2H$ (0.07 M) to form $[H_2BNMe_2]_2$ using **1** as the catalyst in 1,2-$F_2C_6H_4$ solvent) has been previously reported and support the observation of induction period from the hydrogen evolution experiment described here.[5]

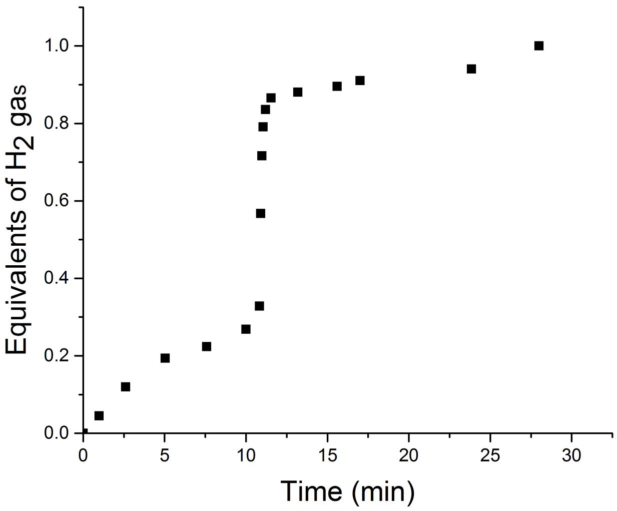

Figure S17 Plot of equivalents of H_2 gas produced vs time during dehydrocoupling of $H_3B{\cdot}NMe_2H$ (0. 41 M) using $[Rh(PPh_2(CH_2)_3PPh_2)(C_6H_5F)][BAr^F_4]$ (**1**, 2×10^{-3} M, 0.5 mol%) in 1,2-$F_2C_6H_4$.

When dehydrocoupling of $H_3B{\cdot}NMe_2H$ in 1,2-$F_2C_6H_4$ was studied in a sealed NMR tube, an induction period of 6 minutes was observed using complex **1** (4 mol%) as the catalyst while no induction period was observed when *in situ* prepared $[\{Rh(PPh_2(CH_2)_3PPh_2)\}_2(\mu\text{-}H)(\mu\text{-}BH_2NMe_2)][BAr^F_4]$ (2 mol%, Rh) was used as the catalyst (Figure S18).

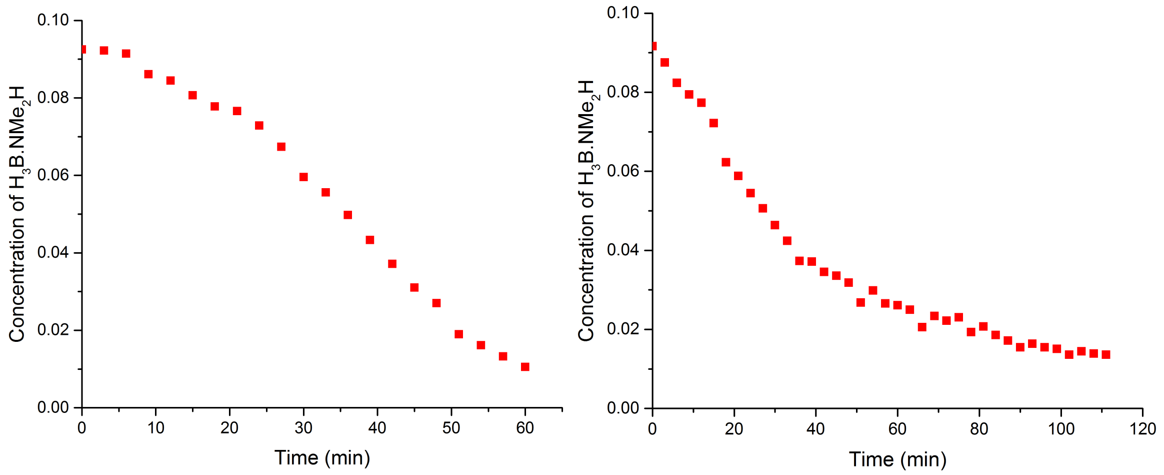

Figure S18 Plots of concentration (M) against time for the catalytic dehydrocoupling of $H_3B{\cdot}NMe_2H$ (9 x 10^{-2} M) in 1,2-$F_2C_6H_4$ (sealed system). Left: **1** (3.6 x 10^{-3} M, 4 mol%); Right: *in situ* prepared [{Rh($PPh_2(CH_2)_3PPh_2$)}$_2$(μ-H)(μ-BH_2NMe_2)][BAr^F_4] (1.8 x 10^{-3} M, 2 mol%).

19. Reactivity of $H_3B{\cdot}NMe_2H$ with 1 in THF: Formation of [{Rh($PPh_2(CH_2)_3PPh_2$)}$_2$(μ-H)(μ-BH_2NMe_2)][BAr^F_4]

1.2 equivalents of $H_3B{\cdot}NMe_2H$ was added to [Rh($PPh_2(CH_2)_3PPh_2$)(C_6H_5F)][BAr^F_4] in THF in a sealed NMR tube. NMR spectroscopy and ESI-MS after 2 minutes showed mixture of complexes, one of which was tentatively characterized as [(Rh($PPh_2(CH_2)_3PPh_2$))$_2$(μ-H)(μ-BH_2NMe_2)][BAr^F_4] (60% yield by $^{31}P\{^1H\}$ NMR spectroscopy). ^1H NMR spectroscopy showed the signal at δ −7.05 (s) presumably due to the bound hydrides (BH_2 and RhH). $^{31}P\{^1H\}$ NMR spectroscopy showed a doublet at δ 23.5 (J_{RhP} = 140). Bound $H_2B{=}NMe_2$ unit was observed at δ 52.7 in ^{11}B NMR spectrum. Signals corresponding to [$BH_2(NMe_2)$(THF)][BAr^F_4] and [$BH_2(NMe_2)_2$][BAr^F_4] were also observed in ^{11}B NMR spectrum as broad triplets at δ 4.8 (t, J_{BH} = 110) and δ −2.8 (t, J_{BH} = 113).[14]

ESI-MS corresponding to [(Rh($PPh_2(CH_2)_3PPh_2$))$_2$(μ-H)(μ-BH_2NMe_2)][BAr^F_4]: m/z 1088.1990 [M+], calc. 1088.1958.

20. Initial rate studies in THF in a closed system

$H_3B{\cdot}NH_2R$ (R = Me and H) and **4** were mixed in a sealed NMR tube to which THF was added. Initial rates at different concentrations of $H_3B{\cdot}NH_2R$ or **4** were calculated from the pseudo zero-order region of the temporal consumption of $H_3B{\cdot}NH_2R$ as measured by ^{11}B NMR spectroscopy over the first 20 minutes of catalysis (~ 5% conversion of $H_3B{\cdot}NH_2R$). Plot of initial rates against concentration of $H_3B{\cdot}NH_2R$ or **4** gives the order of the reaction.[16]

Initial rate studies for $H_3B{\cdot}NH_3$

Table S1 Initial rate data on altering the concentrations of $H_3B{\cdot}NH_3$ and **4**.

[Catalyst] (10^{-3} M)	[$H_3B{\cdot}NH_3$] (10^{-2} M)	Initial rate (10^{-5} Ms^{-1})
1.7	4.6	2.2 ± 0.21
1.7	9.2	3.7 ± 0.62
1.7	18.4	8.0 ± 0.13
0.85	9.2	2.0 ± 0.24
3.4	9.2	8.3 ± 0.41

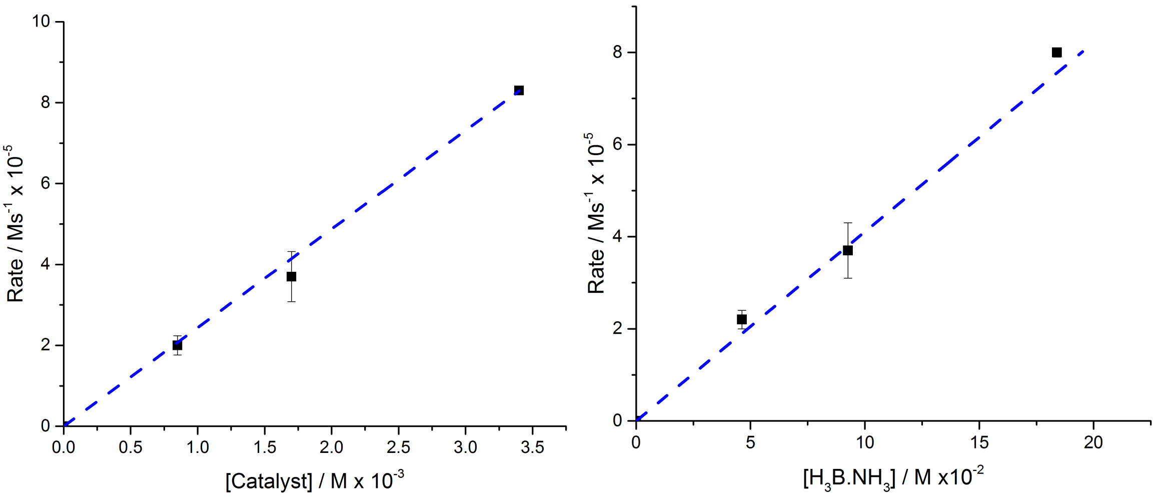

Figure S19 Plot of initial rates against concentrations of **4** (left) and $H_3B{\cdot}NH_3$.

Initial rate studies for $H_3B{\cdot}NH_2Me$

Table S2 Initial rate data on altering the concentrations of $H_3B{\cdot}NH_2Me$ and **4**.

[Catalyst] (10^{-3} M)	[$H_3B{\cdot}NH_2Me$] (10^{-2} M)	Initial rate (10^{-5} Ms^{-1})
1.7	4.5	1.0 ± 0.21
1.7	9.0	1.9 ± 0.62
1.7	18	3.8 ± 0.13
0.85	9.0	0.9 ± 0.11
3.4	9.0	3.5 ± 0.32

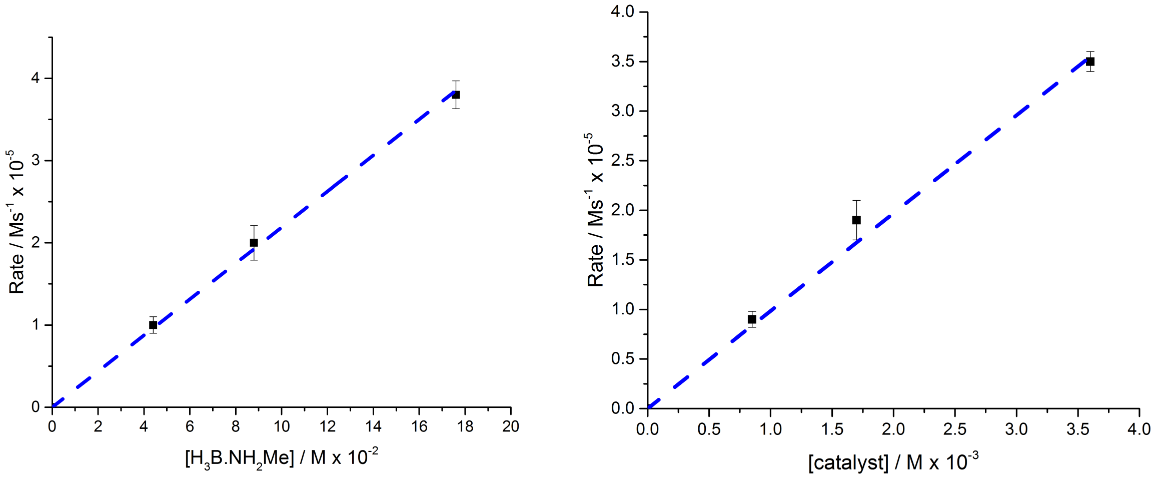

Figure S20 Plot of initial rates against concentrations of **4** (left) and $H_3B{\cdot}NH_2Me$.

21. X-ray crystallography details

X-ray crystallography data for **3** and **4** were collected on an Agilent SuperNova diffractometer using graphite monochromated Cu Kα radiation (λ = 1.54180 Å) and a low-temperature device [173(2) K];[17] data were collected using COLLECT, reduction and cell refinement was performed using DENZO/SCALEPACK.[18] X-ray crystallography data for **6** and **7** were collected on an Enraf Nonius Kappa CCD diffractometer using graphite monochromated Mo Kα radiation (λ = 0.71073 Å) and a low-temperature device [173(2) K];[17] data were collected using SuperNova, reduction and cell refinement was performed using CrysAlis.[19] The structures were solved by direct methods using Superflip[20] and refined full-matrix least squares on F^2 using CRYSTALS.[21] All non-hydrogen atoms were refined with anisotropic displacement parameters. All hydrogen atoms were placed in calculated positions and refined before applying the riding model unless otherwise stated. Crystallographic data have been deposited with the Cambridge Crystallographic Data Centre under CCDC 1438000-1438003. These data can be obtained free of charge from The Cambridge Crystallographic Data Centre via www.ccdc.cam.ac.uk/data_request/cif. Full bond length and bond angle data may be found in the CIFs.

Special refinement details:

Compound 3

Rotational disorder of the CF_3 groups of the anion was treated by modelling the fluorine atoms over two sites and restraining their geometry. One molecule of THF was located in the Fourier map. Due to disorder this solvent molecule could not be adequately modelled and so was treated using the SQUEEZE algorithm[22] leaving a void from which the electron density

was removed. Hydrogen atoms H24, H25, H29 and H30 were located on the Fourier map and allowed to refine freely before using the riding model. H15 was located on the Fourier map and allowed to refine freely without constraints. All other hydrogen atoms were placed in calculated positions and constrained using the riding model.

Compound 4

Rotational disorder of the CF_3 groups of the anion was treated by modelling the fluorine atoms over two sites and restraining their geometry. Hydrogen atoms H1, H2, H3 and H4 were located on the Fourier map and allowed to refine freely before using the riding model. H3 was located on the Fourier map and allowed to refine freely without constraints. All other hydrogen atoms were placed in calculated positions and constrained using the riding model. One isopropyl group showed large displacement ellipsoids and so was modelled over two sites, with restraints utilised to maintain a similar geometry in both disorder units. Similarly the back bone (C_3H_6) of one Dipp3 ligand was disordered over two sites (evident in Fourier map) and so was modelled over two sites and restrained so that both disorder units have similar geometry (but no symmetry was enforced). Shift limiting restraints were used to help convergence to a minimum at the end of refinement.

Compound 6

Rotational disorder of the CF_3 groups of the anion was treated by modelling the fluorine atoms over two sites and restraining their geometry.

Compound 7

Rotational disorder of the CF_3 groups of the anion was treated by modelling the fluorine atoms over two sites and restraining their geometry. BH_3NMe_3 disorder was treated by modelling the BH_3NMe_3 ligand over two sites and using similarity restraints to maintain sensible geometries. B−H hydrides were placed in calculated positions. All hydrogen atoms were refined freely before applying the riding model. Disorder of the phosphine backbone was modelled by splitting the central backbone carbon over two positions and refining the occupancy of the two positions. Hydrogen atoms were then placed at calculated positions referring to each disorder orientation. A small Q-peak of residual electron density was identified at the end of refinement nearby the rhodium atom, this could be due to a small trace of a twinned component or minor disorder.

Table S3 Crystallographic data for **3**, **4**, **6** and **7**.

Compound	**3**	**4**
CCDC No.	1438000	1438001
Formula	$C_{86}H_{69}B_2F_{24}N_1P_4Rh_2$	$C_{62}H_{85}B_2F_{24}N_1P_4Rh_2$
M	1923.78	1651.64
Crystal System	triclinic	monoclinic
Space Group	*P* -1	*P* 2_1/c
T [K]	150	150
a [Å]	12.9810(2)	18.5806(4)
b [Å]	17.1942(3)	15.9539(3)
c [Å]	21.5379(4)	25.1637(4)
α [deg]	68.0529(17)	90
β [deg]	84.7135(15)	97.5683(19)
γ [deg]	83.5328(15)	90
V [Å^3]	4423.92(14)	7394.37(13)
Z	2	4
Radiation Type	Cu K*α*	Cu K*α*
μ (mm^{-1})	4.526	5.299
θ range [deg]	$2.849 \le \theta \le 76.410$	$3.288 \le \theta \le 74.469$
Reflns collected	51685	31990
R_{int}	0.023	0.025
No. of data/restr/param	18267 / 912 / 1180	14608 / 2729 / 1045
R_1 [I>2σ(I)	0.0432	0.0453
wR_2 [all data]	0.1118	0.1196
GoF	0.9781	0.9941
Largest diff. pk and hole [e/Å^3]	1.94, -1.45	1.57, -1.10
Compound	**6**	**7**
CCDC No.	1438002	1438003
Formula	$C_{67}H_{54}O_2P_6Zn_4$	$C_{50}H_{58}B_1F_{24}N_1P_2Rh_1$
M	2125.23	1315.44
Crystal System	triclinic	monoclinic
Space Group	*P* -1	*P* 2_1/c
T [K]	150	150
a [Å]	12.81750(10)	13.01020(10)
b [Å]	14.3695(2)	18.4069(2)
c [Å]	19.6829(3)	24.2512(2)
α [deg]	71.2073(5)	90
β [deg]	76.2971(5)	92.6115(4)
γ [deg]	88.5829(6)	90
V [Å^3]	3329.03(5)	5801.58(5)
Z	2	4
Radiation Type	Mo K*α*	Mo K*α*
μ (mm^{-1})	1.149	0.461
θ range [deg]	$5.133 \le \theta \le 27.513$	$5.099 \le \theta \le 27.485$
Reflns collected	61277	73207
R_{int}	0.024	0.022
No. of data/restr/param	15170 / 852 / 985	13193 / 684 / 833
R_1 [I>2σ(I)	0.0454	0.0736

wR_2 [all data]	0.1154	0.1670
GoF	0.9240	0.9672
Largest diff. pk and hole [e/Å^3]	0.88, -0.65	2.52, -4.45

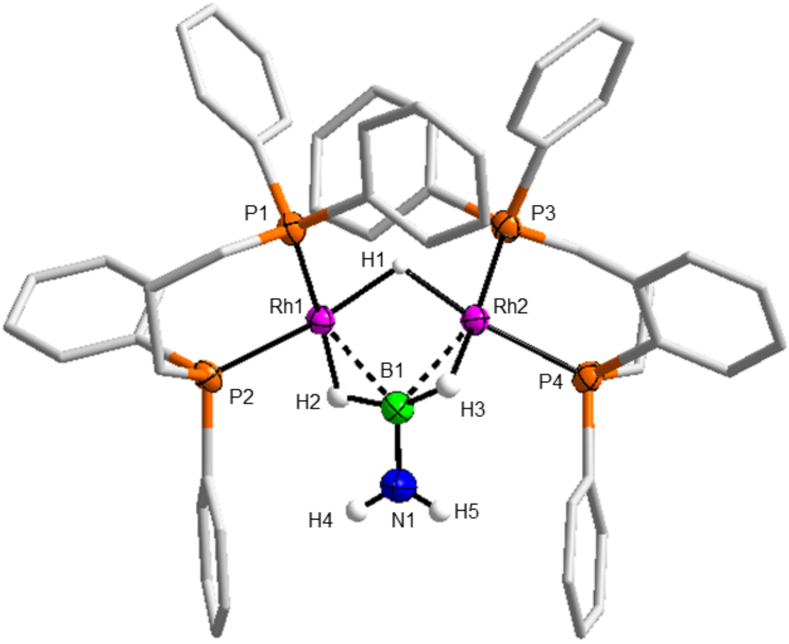

Figure S21 Solid state structure of the cationic portion of **3** with displacement ellipsoids at the 50% probability level. For clarity carbon-bound H atoms are omitted, and the carbon atoms are depicted as a wireframe. Selected bond lengths (Å) and bond angles (˚): Rh1−P1, 2.2928(8); Rh1−P2, 2.2464(8); Rh2−P3, 2.2961(8); Rh2−P4, 2.2544(8); Rh1−B1, 2.111(4); Rh2−B1, 2.077(4); Rh1−Rh2, 2.7905(3); B1−N1, 1.379(5); Rh1−B1−Rh2, 83.55(14).

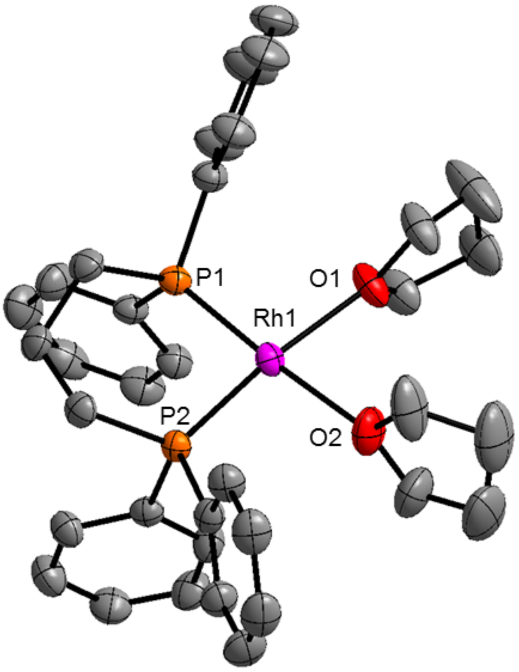

Figure S22 Solid state structure of the cationic portion of **6** with displacement ellipsoids at the 50% probability level. For clarity carbon-bound H atoms are omitted. Selected bond lengths (Å): Rh1−P1, 2.1723(7); Rh1−P2, 2.1775(7); Rh1−O1, 2.181(2); Rh1−O2, 2.176(2), Selected bond angles (˚) O1−Rh1−O2, 81.80 (9).

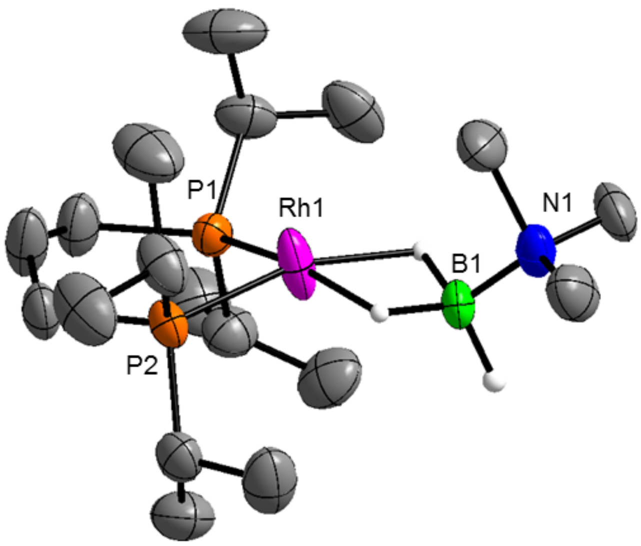

Figure S23 Solid state structure of the cationic portion of **7** with displacement ellipsoids at the 50% probability level. For clarity carbon-bound H atoms are omitted, and only one disordered component is shown. Selected bond lengths (Å): Rh1−P1, 2.2085(11); Rh1−P2, 2.2013(10); Rh1−B1, 2.283(9); B1−N1, 1.535(5), Selected bond angle (˚) P1−Rh1−P2, 93.56(4).

22. Computational details and references

Calculations were run with Gaussian 03 Revision D.01[a] with PCM solvent corrections run with Gaussian 09, Revision D.01.[b] Geometry optimisations were performed using the BP86 functional[c] with Rh and P centres described with the Stuttgart RECPs and associated basis sets[d] (with added d-orbital polarisation on P (ζ = 0.387)[e] and 6-31G** basis sets[f] for all other atoms (called BS1). All stationary points were fully characterized via analytical frequency calculations as either minima (all positive eigenvalues) or transition states (one negative eigenvalue) and IRC calculations and subsequent geometry optimizations were used to confirm the minima linked by the fluxionality transition states. Frequency calculations also provided a free energy in the gas-phase, computed at 298.15 K and 1 atm. Energies reported in the text are based on the gas-phase free energies and incorporate a correction for dispersion effects using Grimme's D3 parameter set[g] (i.e. BP86-D3) as well as solvation (PCM approach) in THF.

Atoms in Molecules[h] analyses were performed with the AIMALL program[i] and employed partially optimized structures based on the experimental heavy atom positions with fully optimized H atoms positions. NMR chemical shifts were calculated with the B3LYP functional[j] with Stuttgart RECPs and basis sets on Rh and P (with d-orbital polarisation on the latter, as above) and 6-311g++** basis sets[k] on C, H, O, B and N (BS2) and were based on the BP86-optimised geometries. Computed chemical shifts are quoted relative to $BF_3{\cdot}OEt_2$. All geometries are provided as sets of Cartesian coordinates as well as the separate file amino-borane.xyz readable by Chemcraft[l] and Mercury.[m] Pipek-Mezey localised orbitals were computed with ORCA Version 3.0.3[n] using def2-TZVP basis sets[o] on the atoms of the {Rh(μ-H)(μ-amino-borane)Rh} unit with a [SD(28,MHF)] ECP for Rh[p] and def2-SV(P) on other atoms.[q]

References

a. Gaussian 03, Revision D.01, M. J. Frisch, G. W. Trucks, H. B. Schlegel, G. E. Scuseria, M. A. Robb, J. R. Cheeseman, J. A. Montgomery, Jr., T. Vreven, K. N. Kudin, J. C. Burant, J. M. Millam, S. S. Iyengar, J. Tomasi, V. Barone, B. Mennucci, M. Cossi, G. Scalmani, N. Rega, G. A. Petersson, H. Nakatsuji, M. Hada, M. Ehara, K. Toyota, R. Fukuda, J. Hasegawa, M. Ishida, T. Nakajima, Y. Honda, O. Kitao, H. Nakai, M. Klene, X. Li, J. E. Knox, H. P. Hratchian, J. B. Cross, C. Adamo, J. Jaramillo, R. Gomperts, R. E. Stratmann, O. Yazyev, A. J. Austin, R. Cammi, C. Pomelli, J. W. Ochterski, P. Y. Ayala, K. Morokuma, G. A. Voth, P. Salvador, J. J. Dannenberg, V. G. Zakrzewski, S. Dapprich, A. D. Daniels, M. C. Strain, O. Farkas, D. K. Malick, A. D. Rabuck, K. Raghavachari, J. B. Foresman, J. V. Ortiz, Q. Cui, A. G. Baboul, S. Clifford, J. Cioslowski, B. B. Stefanov, G. Liu, A. Liashenko, P. Piskorz, I. Komaromi, R. L. Martin, D. J. Fox, T. Keith, M. A. Al-Laham, C. Y. Peng, A. Nanayakkara, M. Challacombe, P. M. W. Gill, B. Johnson, W. Chen, M. W. Wong, C. Gonzalez, and J. A. Pople, Gaussian, Inc., Wallingford CT, 2004.

b. Gaussian 09, Revision D.01, M. J. Frisch, G. W. Trucks, H. B. Schlegel, G. E. Scuseria, M. A. Robb, J. R. Cheeseman, G. Scalmani, V. Barone, B. Mennucci, G. A. Petersson, H. Nakatsuji, M. Caricato, X. Li, H. P. Hratchian, A. F. Izmaylov, J. Bloino, G. Zheng, J. L. Sonnenberg, M. Hada, M. Ehara, K. Toyota, R. Fukuda, J. Hasegawa, M. Ishida, T. Nakajima, Y. Honda, O. Kitao, H. Nakai, T. Vreven, J. A. Montgomery, Jr., J. E. Peralta, F. Ogliaro, M. Bearpark, J. J. Heyd, E. Brothers, K. N. Kudin, V. N. Staroverov, R. Kobayashi, J. Normand, K. Raghavachari, A. Rendell, J. C. Burant, S. S. Iyengar, J. Tomasi, M. Cossi, N. Rega, J. M. Millam, M. Klene, J. E. Knox, J. B. Cross, V. Bakken, C. Adamo, J. Jaramillo, R. Gomperts, R. E. Stratmann, O. Yazyev, A. J. Austin, R. Cammi, C. Pomelli, J. W. Ochterski, R. L. Martin, K. Morokuma, V. G. Zakrzewski, G. A. Voth, P. Salvador, J. J. Dannenberg, S. Dapprich, A. D. Daniels, Ö. Farkas, J. B. Foresman, J. V. Ortiz, J. Cioslowski, and D. J. Fox, Gaussian, Inc., Wallingford CT, 2009.

c. (i) A. D. Becke, *Phys. Rev. A* **1988**, *38*, 3098. (ii) J. P. Perdew, *Phys. Rev. B* **1986**, *33*, 8822.

d. D. Andrae, U. Häußermann, M. Dolg, H. Stoll and H. Preuß, *Theor. Chim. Acta* **1990**, *77*, 123.

e. A. Hollwarth, M. Bohme, S. Dapprich, A. W. Ehlers, A. Gobbi, V. Jonas, K. F. Kohler, R. Stegmann, A. Veldkamp and G. Frenking, *Chem. Phys. Lett.* **1993**, *208*, 237.

f. (i) W. J. Hehre, R. Ditchfield and J. A. Pople, *J. Chem. Phys.* **1972**, *56*, 2257. (ii) P. C. Hariharan and J. A. Pople, *Theor. Chim. Acta.* **1973**, *28*, 213.

g. S. Grimme, J. Antony, S. Ehrlich and H. Krieg, *J. Chem. Phys.* **2010**, *132*, 154104.

h. *Atoms in Molecules - A Quantum Theory* R.F.W. Bader, Oxford University Press, Oxford, **1990**.

i. AIMAll (Version 13.02.26, Professional), Keith, T. A.; Gristmill, T. K. Software, Overland Park KS, USA, 2015 (aim.tkgristmill.com).

i. (i) A. D. Becke, *J. Chem. Phys.,* **1993**, *98*, 5648-52; (ii) C. Lee, W. Yang, and R. G. Parr, *Phys. Rev. B*, **1988**, *37*, 785; (iii) S. H. Vosko, L. Wilk, and M. Nusair, *Can. J. Phys.*, **1980**, *58*,1200-11.

k. A. D. McLean and G. S. Chandler, *J. Chem. Phys.*, **1980**, *72,* 5639.

l Chemcraft version 1.7 see http://www.chemcraftprog.com

m. Mercury CSD 2.0 C. F. Macrae, I. J. Bruno, J. A. Chisholm, P. R. Edgington, P. McCabe, E. Pidcock, L. Rodriguez-Monge, R. Taylor, J. van de Streek and P. A. Wood, J. Appl. Cryst., 41, 466-470, 2008

n. Frank Neese, Wiley Interdisciplinary Reviews: Computational Molecular Science **2012**, *2*, 73.

o. F. Weigend, R. Ahlrichs, Phys. Chem. Chem. Phys. 2005, 7, 3297.

p. Dirk Andrae, Diplomarbeit (1989) (unpublished).Your calculation utilizes the basis: Ahlrichs-VDZ

q. A. Schaefer, H. Horn and R. Ahlrichs, *J. Chem. Phys*. **1992**, *97*, 2571; (ii) A. Schaefer, C. Huber and R. Ahlrichs, *J. Chem. Phys.* **1994**, *100*, 5829.

23. QTAIM results

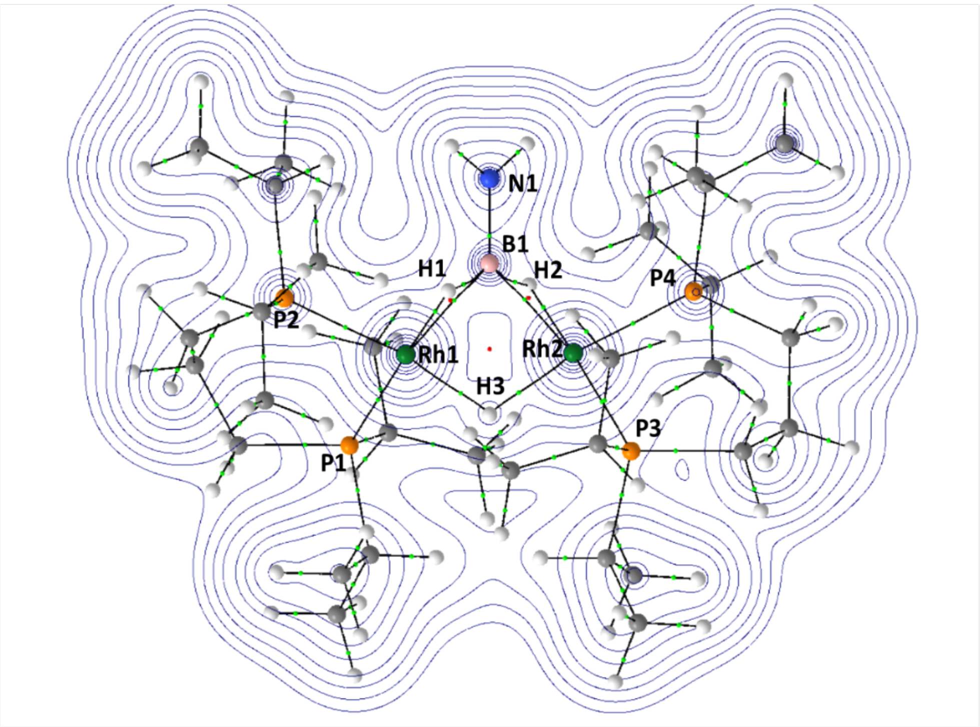

Figure S24 Contour plots of the electron density of **4** presented in the {Rh1H1Rh2} plane with projected stationary points and bond paths. Bond critical points (BCP) are shown in green and ring critical points (RCP) in red. Weak bond paths and chemically less–relevant RCPs are omitted for clarity.

Table S4 Comparison of key bond distances (Å) between the experimental and computed structures of **4**.

	Experiment	Computed
**B1 – Rh1**	2.070(5)	2.076
**B1 – Rh2**	2.055(5)	2.075
**B1 – H1**	1.358	1.476
**B1 – H2**	1.485	1.478
**H1 – Rh1**	1.717	1.660
**H2 – Rh2**	1.724	1.662
**H3 – Rh1**	1.663	1.750
**H3 – Rh2**	1.737	1.749
**P1 – Rh1**	2.2550(10)	2.329
**P2 – Rh1**	2.3063(10)	2.358
**B1 – N1**	1.377(6)	1.422
**Rh1 ··· Rh2**	2.787(4)	2.847

Table S5 Calculated QTAIM parameters (a.u.) for selected BCPs in **4**. (*ρ(r)* = electron density, $\nabla^2 \rho(r)$ = Laplacian of electron density, ε = bond ellipticity, H(*r*) = local energy density, V(*r*) = potential energy density, G(*r*) = kinetic energy density).

	ρ(r)	$\nabla^2 \rho(r)$	***ε***	***H(r)***	***V(r)***	***G(r)***
**B1 – Rh1**	0.11	0.02	0.39	-0.05	-0.11	0.06
**B1 – Rh2**	0.11	0.03	0.54	-0.05	-0.11	0.06
**B1 – H1**	0.11	-0.14	0.36	-0.07	-0.10	0.03
**B1 – H2**	0.11	-0.13	0.37	-0.07	-0.10	0.03
**Rh1 – H1**	0.11	0.25	0.52	-0.04	-0.14	0.10
**Rh2 – H2**	0.11	0.26	0.52	-0.04	-0.15	0.11
**Rh1 – H3**	0.09	0.16	0.12	-0.03	-0.10	0.07
**Rh2 – H3**	0.09	0.16	0.11	-0.03	-0.10	0.07
**B1 – N1**	0.20	0.69	0.14	-0.16	-0.49	0.33

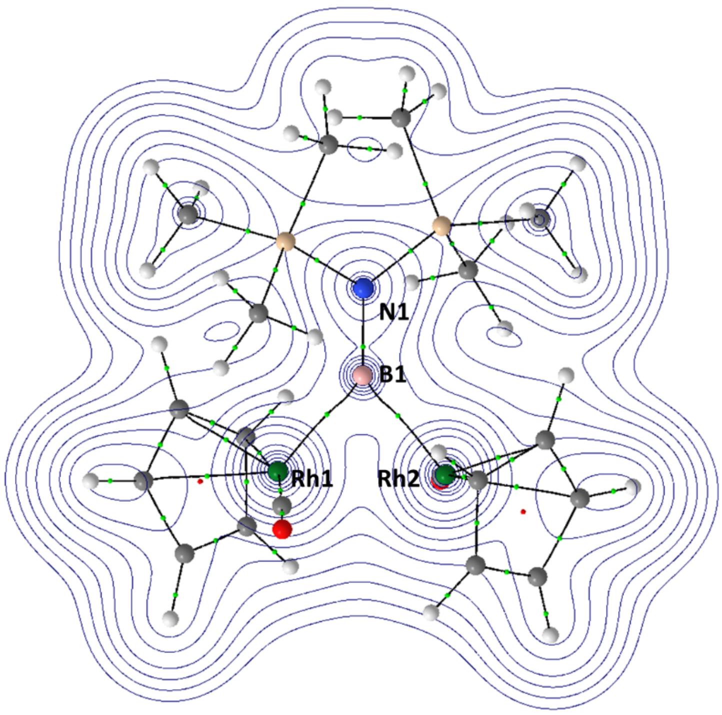

Figure S25 Contour plots of the electron density of **C** presented in the {Rh1H1Rh2} plane with projected stationary points and bond paths. Bond critical points (BCP) are shown in green and ring critical points (RCP) in red. Weak bond paths and chemically less-relevant non-important RCPs are omitted for clarity.

Table S6 Comparison of key bond distances (Å) between the experimental and computed structures of **C**.

	**Experiment**	**Computed**
**B1 – Rh1**	2.054(2)	2.079
**B1 – Rh2**	2.054(2)	2.079
**B1 – N1**	1.399(3)	1.410
**Rh1 ··· Rh2**	2.668(3)	2.686

Table S7 Calculated QTAIM parameters (a.u) for selected BCPs in **C**. (*ρ(r)* = electron density, $\nabla^2 \rho(r)$ = Laplacian of electron density, ε = bond ellipticity, H(*r*) = local energy density, V(*r*) = potential energy density, G(*r*) = kinetic energy density).

	ρ(r)	$\nabla^2 \rho(r)$	***ε***	***H(r)***	***V(r)***	***G(r)***
**B1 – Rh1**	0.11	-0.03	0.08	-0.06	-0.11	0.05
**B1 – Rh2**	0.11	-0.03	0.08	-0.06	-0.11	0.05
**B1 – N1**	0.21	0.64	0.10	-0.17	-0.50	0.33

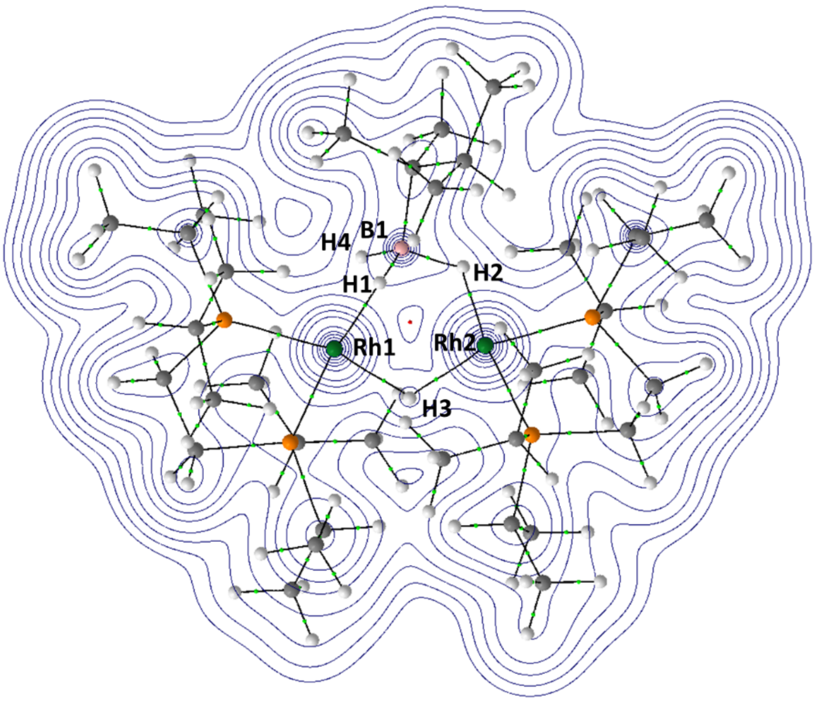

Figure S26 Contour plots of the electron density of **D** presented in the {Rh1H1Rh2} plane with projected stationary points and bond paths. Bond critical points (BCP) are shown in green and ring critical points (RCP) in red. Weak bond paths and chemically less-relevant RCPs are omitted for clarity.

Table S8 Comparison of key bond distances (Å) between the experimental and computed structures of **D**.

	Experiment	Computed
**B1 – H1**	1.262(25)	1.337
**B1 – H2**	1.230(27)	1.321
**H1 – Rh1**	1.739(24)	1.803
**H2 – Rh2**	1.787(26)	1.708
**H3 – Rh1**	1.722(26)	1.741
**H3 – Rh2**	1.755(25)	1.771
**B1 – H4**	1.175(23)	1.248
**Rh1 – P**	2.2154(7)	2.259
**Rh1 – P**	2.2108(8)	2.290
**Rh2 – P**	2.2380(7)	2.272
**Rh2 – P**	2.2026(7)	2.267
**Rh1 – Rh2**	2.9120(6)	2.936

Table S9 Calculated QTAIM parameters (a.u.) for selected BCPs in **D**. (*ρ(r)* = electron density, $\nabla^2 \rho(r)$ = Laplacian of electron density, ε = bond ellipticity, H(*r*) = local energy density, V(*r*) = potential energy density, G(*r*) = kinetic energy density).

	ρ(r)	$\nabla^2 \rho(r)$	*ε*	*H(r)*	*V(r)*	*G(r)*
**B1 – Rh1/Rh2**	-	-	-	-	-	-
**B1 – H1**	0.13	-0.10	0.23	-0.11	-0.21	0.09
**B1 – H2**	0.12	-0.07	0.10	-0.10	-0.20	0.09
**B1 – H4**	0.15	-0.20	0.11	-0.15	-0.27	0.11
**Rh1 – H1**	0.09	0.24	0.24	-0.03	-0.12	0.09
**Rh2 – H2**	0.08	0.22	0.50	-0.03	-0.10	0.07
**Rh1 – H3**	0.08	0.15	0.12	-0.02	-0.09	0.07
**Rh2 – H3**	0.09	0.16	0.10	-0.03	-0.10	0.07

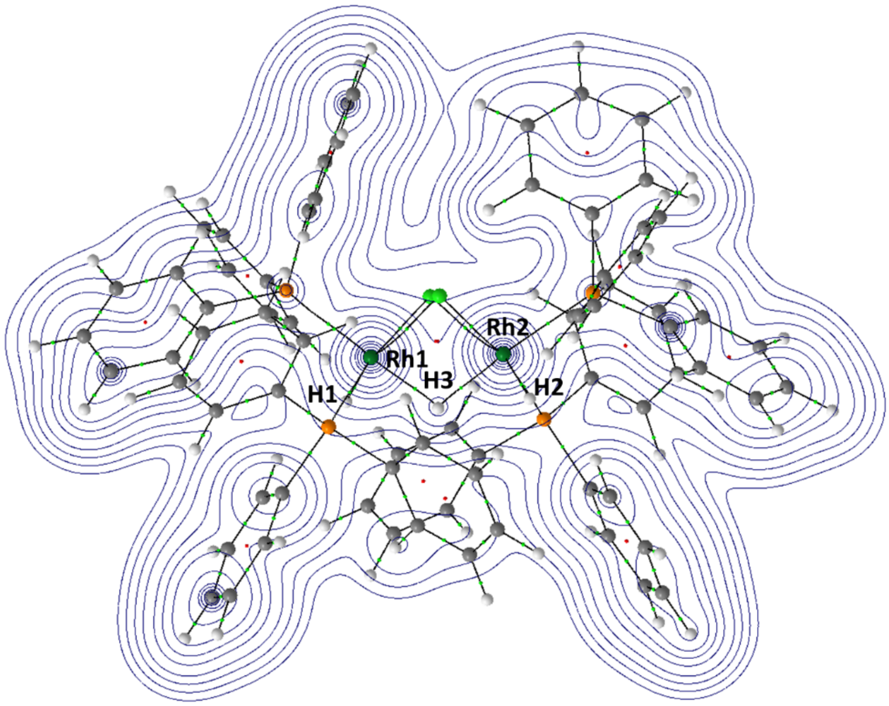

Figure S27 Contour plots of the electron density of **E** presented in the {Rh1H1Rh2} plane with projected stationary points and bond paths. Bond critical points (BCP) are shown in green and ring critical points (RCP) in red. Weak bond paths and chemically less-relevant RCPs are omitted for clarity.

Table S10 Comparison of key bond distances (Å) between the experimental and computed structures of **E**.

	**Experiment**	**Computed**
**H1 – Rh1**	1.59(7)	1.537
**H2 – Rh2**	1.60(9)	1.539
**H3 – Rh1**	1.82(6)	1.762
**H3 – Rh2**	1.82(6)	1.765
**Rh1 – Cl**	2.416(3)	2.469
**Rh1 – Cl**	2.553(3)	2.567
**Rh2 – Cl**	2.394(2)	2.459
**Rh2 – Cl**	2.538(3)	2.600
**Rh1 – P**	2.257(3)	2.305
**Rh 1 – P**	2.313(3)	2.366
**Rh2 – P**	2.256(3)	2.309
**Rh2 – P**	2.301(3)	2.378
**Rh1 ··· Rh2**	2.7758(11)	2.808

Table S11 Calculated QTAIM parameters (a.u.) for selected BCPs in **E**. (*ρ(r)* = electron density, $\nabla^2 \rho(r)$ = Laplacian of electron density, ε = bond ellipticity, H(*r*) = local energy density, V(*r*) = potential energy density, G(*r*) = kinetic energy density).

	ρ(r)	$\nabla^2 \rho(r)$	***ε***	***H(r)***	***V(r)***	***G(r)***
**Rh1 – H1**	0.16	0.06	0.01	-0.10	-0.20	0.10
**Rh2 – H2**	0.16	0.06	0.01	-0.09	-0.19	0.10
**Rh1 – H3**	0.09	0.16	0.12	-0.03	-0.10	0.07
**Rh2 – H3**	0.09	0.16	0.11	-0.03	-0.10	0.07

24. Calculated ^{11}B NMR shifts

Table S12 Calculated ^{11}B NMR shifts (ppm, B3LYP(BS2)///BP86(BS1).

Complex	^{11}B shift
**4**	51.04
**C**	97.53

25. Pipek-Mezey localised orbitals

(a)

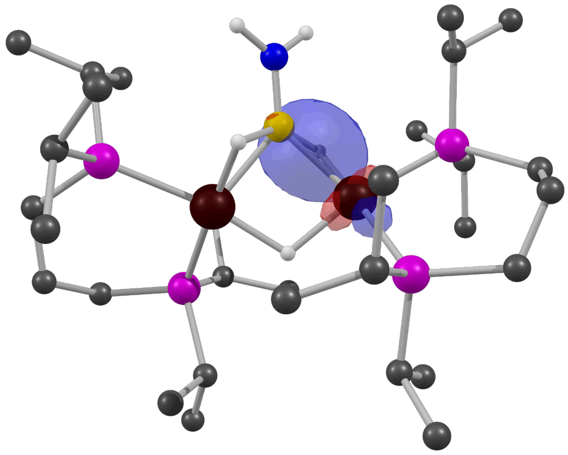

(b)

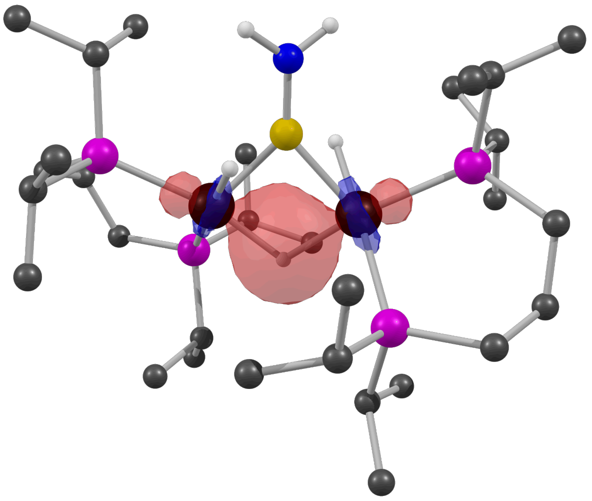

Figure S28 Pipek-Mezey localised orbitals for (a) the {Rh2-H2-B1} interaction (cf. {Rh1-H1-B1} interaction shown in **Figure 3**); (b) the {Rh1-H3-Rh2} interaction.

26. Cartesian coordinates (Å) and computed energies (in hartrees) for all optimised structures. All structures can be visualized via the amino-borane.xyz file provided.

```
4
SCF Energy = -1514.46765082
Enthalpy 0K = -1513.503193
Enthalpy 298K = -1513.443864
Free Energy 298K = -1513.592384
SCF(THF) = -1514.50380934
SCF(BP86-D3) = -0.21807101
Lowest Frequencies = 23.2682
26.9424 cm-1
B   0.01925 -1.67196  0.03586
C  -4.13365  1.39013  1.72672
C  -5.00426  1.03774  0.50357
C  -5.03774 -0.46483  0.16912
C   4.07331  1.45389 -1.81697
C   4.82132  0.11141 -1.70802
C   5.02330 -0.38401 -0.26275
C  -1.58364  1.24017  3.16225
C  -0.11569  1.68916  3.24947
C  -1.74984 -0.20558  3.66189
C  -1.92009  3.22101  0.93166
C  -2.37280  4.26574  1.96614
C  -2.46734  3.54256 -0.47191
C  -3.61918 -3.03648 -0.03143
C  -3.32446 -3.20244  1.46932
C  -4.95450 -3.69234 -0.42946
C  -3.89080 -1.26545 -2.41273
C  -3.98707  0.15415 -2.99993
C  -2.90400 -2.13411 -3.21053
C   4.02394 -0.91895  2.42825
C   3.99514  0.55476  2.87191
C   3.15192 -1.78290  3.35630
C   3.65878 -2.96631  0.28276
C   3.39804 -3.29187 -1.19962
C   4.96746 -3.60770  0.77834
C   1.44864  1.26122 -3.13831
C  -0.04011  1.63718 -3.13917
C   1.67308 -0.15544 -3.69542
C   1.90782  3.24152 -0.92962
C   2.48832  3.53066  0.46709
C   2.37760  4.27226 -1.97136
H  -4.41446  2.37968  2.12667
H  -4.31335  0.66061  2.53706
H  -0.63673 -3.66066 -0.46946
H   0.71466 -3.64240  0.56321
H  -4.67834  1.61752 -0.37833
H  -6.04123  1.36118  0.71221
H  -5.84852 -0.67154 -0.55166
H  -5.27803 -1.03508  1.08502
H   4.17483  1.86023 -2.83884
H   4.54377  2.18747 -1.13681
H   4.31747 -0.66231 -2.31326
H   5.82056  0.24579 -2.16350
H   5.39659  0.44664  0.36334
H   5.80203 -1.16561 -0.23735
H  -2.21080  1.91308  3.77971
H   0.26565  1.51361  4.27107
H   0.51191  1.10981  2.54364
H   0.01631  2.76116  3.02787
H  -1.14439 -0.89391  3.04829
H  -1.41368 -0.28271  4.71102
H  -2.79638 -0.55105  3.62385
H  -0.81476  3.22179  0.87896
H  -3.47276  4.32314  2.03670
H  -1.97628  4.07007  2.97563
H  -2.02190  5.26800  1.66218
H  -2.09364  4.52518 -0.81145
H  -3.56944  3.60225 -0.47145
H  -2.16389  2.78362 -1.21302
H  -2.80469 -3.52812 -0.59746
H  -3.32773 -4.27370  1.73832
H  -2.34331 -2.77796  1.73380
H  -4.09516 -2.71381  2.09137
H  -5.14602 -3.65139 -1.51409
H  -5.80904 -3.22319  0.08689
H  -4.94634 -4.75741 -0.13713
H  -4.89425 -1.73040 -2.45787
H  -4.26545  0.10186 -4.06761
H  -3.01911  0.67922 -2.92822
H  -4.74968  0.76792 -2.49234
H  -1.87689 -1.73663 -3.13753
H  -3.18769 -2.13897 -4.27789
H  -2.89312 -3.18372 -2.87146
H   5.06825 -1.28418  2.46477
H   4.36626  0.64278  3.90847
H   2.96526  0.95066  2.84573
H   4.62534  1.20572  2.24317
H   3.48260 -1.66042  4.40278
H   2.09402 -1.47277  3.30051
H   3.20825 -2.85804  3.11896
H   2.82422 -3.37767  0.88325
H   3.34285 -4.38573 -1.34293
H   2.45361 -2.84692 -1.55222
H   4.21987 -2.92539 -1.83888
H   5.14366 -3.45363  1.85477
H   5.84721 -3.22946  0.22973
H   4.93148 -4.69786  0.60454
H   2.00495  1.98687 -3.76336
H  -0.46095  1.48318 -4.14888
H  -0.60838  1.00394 -2.42738
H  -0.20715  2.69307 -2.86917
H   1.21333 -0.90861 -3.03294
H   1.21482 -0.24393 -4.69634
```

```
H   2.74277 -0.39954 -3.80037
H   0.80421  3.27603 -0.85566
H   2.19069  4.54002  0.80281
H   2.13484  2.79891  1.21323
H   3.59255  3.50729  0.46480
H   3.47478  4.26341 -2.08875
H   2.09605  5.28975 -1.64678
H   1.92728  4.10775 -2.96409
H  -0.00817  0.85534  0.01893
H  -0.74762 -1.28054 -1.16246
H   0.79638 -1.24964  1.21959
N   0.03038 -3.09340  0.04825
P   3.50113 -1.08876  0.60171
P   2.23139  1.42788 -1.40944
P  -2.28079  1.40892  1.39954
P  -3.45647 -1.20706 -0.55552
Rh  1.42063 -0.14500  0.14618
Rh -1.41416 -0.17770 -0.11557
```

TS(4-4')
SCF Energy = -1514.45536401
Enthalpy 0K = -1513.490911
Enthalpy 298K = -1513.433261
Free Energy 298K = -1513.576420
SCF(THF) = -1514.49211422
SCF(BP86-D3) = -0.21234476
Lowest Frequencies = -46.9194
21.6696 cm-1

```
B  -0.17759 -1.01049  1.30881
C  -4.71802  1.01330  0.48397
C  -4.89680  0.33592 -0.89429
C  -4.63417 -1.18404 -0.90596
C   4.13905  1.63715 -1.52400
C   4.97633  0.35147 -1.39392
C   5.11062 -0.14711  0.05386
C  -3.13124  1.58044  2.90562
C  -1.82561  2.10542  3.52972
C  -3.63652  0.33376  3.64930
C  -2.62214  3.12181  0.42679
C  -3.53673  4.20231  1.02936
C  -2.64472  3.17568 -1.11264
C  -2.85551 -3.33781  0.11045
C  -3.51221 -3.14135  1.49087
C  -3.52492 -4.51053 -0.63000
C  -2.54726 -2.25509 -2.66096
C  -2.75022 -1.08716 -3.64141
C  -1.17472 -2.92085 -2.84904
C   3.92243 -0.42289  2.69327
C   3.78072  1.08281  2.97287
C   3.04328 -1.23091  3.66212
C   3.79056 -2.71253  0.75666
C   3.68135 -3.17633 -0.70829
C   5.06972 -3.25041  1.42387
C   1.73964  1.26606 -3.16680
C   0.24899  1.58451 -3.35473
C   2.09481 -0.12308 -3.72647
C   1.71491  3.20186 -0.89571
C   2.06238  3.54145  0.56435
C   2.22932  4.28461 -1.86292
H  -5.24777  1.98359  0.49723
H  -5.19689  0.38807  1.25965
H  -1.42993 -1.55029  2.96721
H   0.16981 -2.12348  3.11117
H  -4.27001  0.83653 -1.65599
H  -5.94494  0.50026 -1.21245
H  -5.08155 -1.63623 -1.81025
H  -5.14019 -1.64789 -0.04008
H   4.31844  2.11015 -2.50644
H   4.46420  2.36390 -0.75551
H   4.57182 -0.44866 -2.03987
H   5.99348  0.56507 -1.77675
H   5.48143  0.67678  0.69164
H   5.86711 -0.95012  0.10793
H  -3.91315  2.36027  2.98563
H  -1.97389  2.29195  4.60959
H  -1.00903  1.36999  3.42013
H  -1.49184  3.05302  3.07385
H  -2.87637 -0.46575  3.65519
H  -3.84208  0.58918  4.70519
H  -4.56643 -0.08238  3.22593
H  -1.58206  3.30519  0.75708
H  -4.60184  4.03074  0.78907
H  -3.44149  4.28422  2.12517
H  -3.26979  5.18853  0.60531
H  -2.30385  4.16728 -1.46497
H  -3.66369  3.02290 -1.51134
H  -1.98044  2.41305 -1.56053
H  -1.78384 -3.56443  0.26569
H  -3.23831 -3.97400  2.16415
H  -3.20726 -2.19579  1.96756
H  -4.61456 -3.13763  1.41512
H  -2.99561 -4.79865 -1.55287
H  -4.57427 -4.28086 -0.89081
H  -3.54164 -5.39996  0.02772
H  -3.34341 -3.00311 -2.84167
H  -2.61949 -1.44055 -4.68120
H  -2.01046 -0.28673 -3.46696
H  -3.75936 -0.64469 -3.56967
H  -0.35913 -2.21343 -2.61620
H  -1.05477 -3.25193 -3.89741
H  -1.04203 -3.80649 -2.20267
H   4.98176 -0.71766  2.83259
H   4.09220  1.30497  4.01053
H   2.73020  1.40014  2.85239
H   4.40007  1.70487  2.30305
H   3.33450 -1.01103  4.70592
H   1.98236 -0.94499  3.54805
H   3.13483 -2.32181  3.51799
H   2.91143 -3.10522  1.30481
H   3.68818 -4.28106 -0.75881
H   2.75102 -2.81034 -1.17721
H   4.53633 -2.81708 -1.31066
H   5.14619 -2.98293  2.49155
H   5.98447 -2.89313  0.91729
```

```
H   5.08239 -4.35437  1.35857
H   2.33753  2.02822 -3.70446
H  -0.04788  1.39830 -4.40370
H  -0.37199  0.93781 -2.70559
H   0.01020  2.63644 -3.12284
H   1.58700 -0.91992 -3.15483
H   1.77198 -0.19640 -4.78198
H   3.17967 -0.32310 -3.70058
H   0.61263  3.13145 -0.97372
H   1.64118  4.52903  0.83051
H   1.65598  2.78774  1.25961
H   3.15470  3.60400  0.72404
H   3.32999  4.37149 -1.82933
H   1.81880  5.26905 -1.57086
H   1.93319  4.10370 -2.91044
H  -0.08964  1.07173  0.39885
H  -0.00718 -0.60275 -1.02972
H   1.00935 -1.60649  0.95320
N  -0.49833 -1.58645  2.55670
P   3.54056 -0.81731  0.86221
P   2.27324  1.43564 -1.34186
P  -2.92901  1.32428  1.02313
P  -2.80547 -1.68717 -0.86004
Rh  1.44701 -0.21202  0.09662
Rh -1.28146 -0.02293 -0.04097
```

4'
SCF Energy = -1514.46326402
Enthalpy 0K = -1513.499211
Enthalpy 298K = -1513.439646
Free Energy 298K = -1513.588930
SCF(THF) = -1514.49876963
SCF(BP86-D3) = -0.21595541
Lowest Frequencies = 20.5868
23.7903 cm-1

```
B   0.09677  0.38535  1.43887
C   4.92484  0.48253  0.58094
C   4.94825  1.14167 -0.81619
C   3.96507  2.31781 -0.98494
C  -4.02011 -2.37670 -0.74048
C  -4.99839 -1.18586 -0.77082
C  -5.00661 -0.34960  0.52161
C   3.81893 -1.23981  2.68603
C   2.79741 -2.29146  3.15432
C   3.86415 -0.02071  3.62853
C   4.04160 -2.31124 -0.07434
C   5.54112 -2.64566 -0.02318
C   3.50554 -2.28017 -1.51873
C   1.36358  3.49580 -0.24944
C   1.72775  3.62223  1.24164
C   1.70608  4.78615 -1.01622
C   1.72551  2.08644 -2.86042
C   2.38101  0.94056 -3.65173
C   0.21145  2.15753 -3.11074
C  -3.75884  1.16684  2.65313
C  -3.39881 -0.04344  3.53539
C  -2.99341  2.43454  3.07216
C  -3.95578  2.39837 -0.06038
C  -3.76686  2.28962 -1.58489
C  -5.33537  2.97331  0.30310
C  -1.86243 -2.30738 -2.71090
C  -0.36560 -2.41994 -3.03586
C  -2.55245 -1.24866 -3.59002
C  -1.36950 -3.43604  0.02478
C  -1.62208 -3.35779  1.54157
C  -1.75949 -4.81016 -0.54804
H   5.87335 -0.04897  0.77335
H   4.83661  1.26862  1.35133
H   1.01821  0.15108  3.35595
H  -0.51014  0.90408  3.43619
H   4.78801  0.38732 -1.60759
H   5.96819  1.53464 -0.98666
H   4.19099  2.85794 -1.92155
H   4.12815  3.03723 -0.16226
H  -4.25237 -3.07548 -1.56348
H  -4.16201 -2.93855  0.20082
H  -4.79695 -0.54146 -1.64452
H  -6.01782 -1.58760 -0.92272
H  -5.07643 -1.02302  1.39470
H  -5.90027  0.29776  0.55348
H   4.82307 -1.70643  2.68081
H   3.03159 -2.60351  4.18731
H   1.76620 -1.89971  3.13756
H   2.80820 -3.19767  2.52713
H   2.96748  0.61621  3.53033
H   3.92303 -0.35963  4.67759
H   4.74148  0.61933  3.44481
H   3.48026 -3.09534  0.46792
H   6.14210 -1.91981 -0.59774
H   5.93784 -2.67914  1.00570
H   5.71858 -3.63965 -0.47098
H   3.61880 -3.27137 -1.99365
H   4.04704 -1.55234 -2.14534
H   2.42882 -2.01844 -1.53834
H   0.27664  3.30283 -0.32163
H   1.20012  4.48780  1.68082
H   1.44936  2.72376  1.81532
H   2.80803  3.80431  1.38435
H   1.36864  4.76627 -2.06486
H   2.79084  4.99076 -1.00990
H   1.21393  5.64771 -0.53050
H   2.19211  3.03962 -3.17453
H   2.17149  1.05364 -4.73027
H   1.98659 -0.04097 -3.33547
H   3.47752  0.92243 -3.53145
H  -0.30038  1.28246 -2.67101
H   0.00503  2.17189 -4.19590
H  -0.24162  3.06323 -2.67466
H  -4.84410  1.36159  2.75506
H  -3.58990  0.19536  4.59663
H  -2.33709 -0.32216  3.42657
H  -3.99700 -0.93676  3.29053
H  -3.05373  2.56762  4.16681
H  -1.92517  2.38340  2.79515
```

```
H  -3.40670  3.34578  2.61085
H  -3.17103  3.07559  0.32668
H  -3.84069  3.28915 -2.04927
H  -2.77915  1.86348 -1.83341
H  -4.54406  1.65813 -2.04933
H  -5.49094  3.06574  1.39047
H  -6.15564  2.36030 -0.10833
H  -5.44406  3.98314 -0.13098
H  -2.34201 -3.28775 -2.89677
H  -0.23080 -2.62350 -4.11312
H   0.16099 -1.47943 -2.79910
H   0.12635 -3.23390 -2.47833
H  -2.16788 -0.23733 -3.37184
H  -2.35991 -1.45963 -4.65695
H  -3.64616 -1.23595 -3.45197
H  -0.29296 -3.24777 -0.14512
H  -1.04564 -4.14644  2.05696
H  -1.31850 -2.37822  1.94567
H  -2.68556 -3.52203  1.79188
H  -2.83924 -5.01050 -0.43684
H  -1.22660 -5.60470  0.00411
H  -1.49923 -4.92253 -1.61314
H   0.99174 -1.56468  0.72124
H  -0.02591 -0.19200 -0.95911
H  -0.80633  1.38672  0.90628
N   0.22620  0.52819  2.84178
P  -3.50976  0.75567  0.80820
P  -2.18121 -1.96185 -0.86482
P   3.51984 -0.73300  0.87308
P   2.11397  1.90911 -1.00271
Rh -1.40744  0.05531  0.07224
Rh  1.41005 -0.18527  0.03064
```

C

SCF Energy = -1162.26077543

Enthalpy 0K = -1161.851573

Enthalpy 298K = -1161.815930

Free Energy 298K = -1161.917595

Lowest Frequencies = 24.0963 27.4491 cm-1

```
C   2.90591  2.75181  0.70632
C   1.78588  3.51316  0.19683
C   0.64023  3.25912  1.03082
C   1.02669  2.26620  1.98815
C   2.43291  1.97302  1.79291
Rh  1.17176  1.33757 -0.11904
B  -0.41601 -0.00000 -0.00001
N  -1.82602 -0.00001 -0.00001
Si -2.77306  1.42953 -0.65987
C  -1.81489  2.46550 -1.92385
Rh  1.17178 -1.33755  0.11904
C   1.22961 -1.08202  1.92527
O   1.27301 -0.96348  3.09791
C   1.78593 -3.51314 -0.19682
C   0.64028 -3.25911 -1.03082
C   1.02675 -2.26619 -1.98815
C   2.43295 -1.97299 -1.79289
C   2.90596 -2.75178 -0.70630
C   1.22961  1.08203 -1.92527
O   1.27303  0.96349 -3.09791
Si -2.77305 -1.42956  0.65987
C  -1.81486 -2.46552  1.92385
C  -3.31852 -2.54192 -0.78352
C  -4.29545 -0.80513  1.62447
C  -3.31854  2.54189  0.78351
C  -4.29546  0.80509 -1.62447
H  -0.33469  3.73689  0.94578
H   3.91498  2.74374  0.29497
H   0.39783  1.84696  2.77287
H   3.01454  1.25860  2.37440
H   1.82362  4.21918 -0.63404
H  -5.00821  0.20114 -1.04351
H  -3.98182  0.21800 -2.50427
H  -4.84143  1.68930 -1.99979
H  -0.95332  2.98527 -1.48174
H  -2.51488  3.21563 -2.33591
H  -1.44697  1.85076 -2.76092
H  -4.04059  2.04057  1.44690
H  -3.79355  3.46008  0.39535
H  -2.45057  2.84002  1.39463
H  -0.33463 -3.73690 -0.94580
H   3.91501 -2.74369 -0.29494
H   0.39789 -1.84696 -2.77288
H   3.01458 -1.25856 -2.37438
H   1.82367 -4.21916  0.63405
H  -5.00821 -0.20119  1.04350
H  -3.98181 -0.21804  2.50427
H  -4.84141 -1.68934  1.99979
H  -0.95330 -2.98529  1.48174
H  -2.51485 -3.21564  2.33591
H  -1.44694 -1.85077  2.76091
H  -4.04057 -2.04061 -1.44691
H  -3.79353 -3.46013 -0.39536
H  -2.45054 -2.84006 -1.39464
```

D

SCF Energy = -1617.05696766

Enthalpy 0K = -1615.994059

Enthalpy 298K = -1615.929254

Free Energy 298K = -1616.089664

Lowest Frequencies = 13.3601 25.5936 cm-1

```
Rh -1.42962 -0.07635  0.08200
Rh  1.50175  0.08771  0.11666
P  -3.56673  0.28480  0.76307
P  -2.26447 -1.77638 -1.16501
P   3.62769  0.83230 -0.29351
P   2.49481 -1.81476  0.82195
C  -4.69061 -1.14358  0.19327
C  -3.82388  0.22788  2.65594
C  -3.15885 -1.02274  3.25493
C  -3.28927  1.49639  3.34259
C  -4.57434  1.84274  0.27021
C  -4.71103  1.91010 -1.26078
```

C	-5.94416	1.99573	0.95481
C	-4.17009	-1.77280	-1.10453
C	-1.95580	-1.59020	-3.04189
C	-2.33532	-0.17667	-3.51548
C	-0.49165	-1.89629	-3.39488
C	-1.82653	-3.61485	-0.86208
C	-2.42562	-4.61972	-1.86108
C	-2.16487	-3.99158	0.59087
C	4.92504	-0.38282	0.39264
C	4.13792	0.97403	-2.13115
C	3.94922	-0.36702	-2.85881
C	3.36158	2.09689	-2.83804
C	4.28803	2.48732	0.41481
C	4.04686	2.55943	1.93310
C	5.75301	2.81263	0.06880
C	4.37497	-1.80860	0.51768
C	2.37724	-2.08819	2.71282
C	0.93619	-2.43963	3.11990
C	2.87656	-0.84866	3.47276
C	2.02189	-3.55129	0.16740
C	2.20768	-3.62052	-1.35729
C	2.72698	-4.73021	0.86338
C	-0.20449	3.49222	0.25137
C	-0.84495	3.99413	1.57270
C	1.11820	4.27050	0.06361
C	-1.18977	3.76730	-0.94710
C	-0.57732	3.47085	-2.33144
C	-1.79493	5.19032	-0.97047
B	0.09902	1.87998	0.43525
H	-1.09945	1.42765	0.82033
H	0.58030	1.40585	-0.69947
H	0.85606	1.63115	1.39517
H	0.10861	-0.92870	-0.12341
H	-5.72938	-0.78401	0.08271
H	-4.69139	-1.88382	1.01221
H	-4.91986	0.16468	2.80382
H	-3.33988	-1.06363	4.34508
H	-2.06962	-0.99491	3.07923
H	-3.54293	-1.95918	2.81724
H	-3.44779	1.43087	4.43473
H	-3.78610	2.41634	2.99177
H	-2.20628	1.60808	3.16494
H	-3.91558	2.67352	0.58807
H	-5.15704	2.87440	-1.56489
H	-5.37406	1.11091	-1.64043
H	-3.73184	1.81099	-1.75673
H	-6.44280	2.91506	0.59610
H	-5.87121	2.06894	2.05204
H	-6.61691	1.15231	0.71598
H	-4.55666	-2.79821	-1.24185
H	-4.49956	-1.18369	-1.97714
H	-2.61929	-2.32796	-3.53554
H	-2.12499	-0.07147	-4.59590
H	-1.74985	0.58084	-2.96613
H	-3.40372	0.05028	-3.36415
H	-0.31541	-1.71739	-4.47142
H	-0.21705	-2.94294	-3.18512
H	0.18712	-1.24093	-2.81921
H	-0.72549	-3.62053	-0.97024
H	-2.09216	-5.64486	-1.61432
H	-2.12324	-4.41721	-2.90148
H	-3.52977	-4.62637	-1.82514
H	-1.79260	-5.00539	0.82750
H	-3.25759	-4.00000	0.75871
H	-1.71662	-3.27738	1.30109
H	5.19970	0.01097	1.38598
H	5.83531	-0.34730	-0.23253
H	5.21640	1.22393	-2.12085
H	4.23282	-0.26654	-3.92305
H	2.89515	-0.68953	-2.80504
H	4.57077	-1.16911	-2.42531
H	3.65340	2.15145	-3.90304
H	3.55169	3.08760	-2.39220
H	2.27523	1.90728	-2.79212
H	3.63197	3.23757	-0.06034
H	4.26867	3.57674	2.30415
H	4.70421	1.86372	2.48578
H	3.00253	2.31569	2.18635
H	6.04131	3.77666	0.52703
H	5.92568	2.90471	-1.01599
H	6.44732	2.04839	0.46082
H	4.52724	-2.36212	-0.42463
H	4.88669	-2.37566	1.31610
H	3.04595	-2.94084	2.94105
H	0.85484	-2.51511	4.21987
H	0.60311	-3.40160	2.69498
H	0.23764	-1.65773	2.77232
H	2.79643	-1.01059	4.56376
H	2.27286	0.03511	3.20490
H	3.93196	-0.61779	3.24841
H	0.93876	-3.59991	0.38553
H	1.71477	-4.52136	-1.76741
H	3.27571	-3.68845	-1.63232
H	1.78824	-2.73043	-1.85166
H	2.39247	-5.68646	0.42005
H	2.51576	-4.77904	1.94396
H	3.82283	-4.68119	0.73268
H	-1.86068	3.58172	1.71449
H	-0.92675	5.09693	1.60977
H	-0.23902	3.67899	2.44117
H	1.68362	3.92034	-0.81700
H	1.76421	4.14703	0.94997
H	0.94388	5.35730	-0.06269
H	-2.02966	3.05575	-0.80861
H	-1.35159	3.52762	-3.11802
H	-0.12675	2.46538	-2.37333
H	0.20423	4.20660	-2.59443
H	-2.46020	5.31041	-1.84467
H	-1.01197	5.96638	-1.05309
H	-2.39053	5.41415	-0.07004

E

SCF Energy = -3059.31054031

Enthalpy 0K = -3058.211173

Enthalpy 298K = -3058.132115
Free Energy 298K = -3058.334584
Lowest Frequencies = 11.5815
13.2761 cm-1

C	-5.61380	2.06789	0.29498
C	-4.97056	1.02150	-0.39527
C	-5.67930	-0.17449	-0.64735
C	-7.00820	-0.31627	-0.22404
C	-7.64580	0.73253	0.45965
C	-6.94483	1.91946	0.72026
P	-3.24132	1.18237	-1.08500
C	-3.56853	0.80993	-2.88533
C	-2.65376	0.07679	-3.66735
C	-2.92550	-0.17099	-5.02367
C	-4.10363	0.31253	-5.61208
C	-5.01748	1.04897	-4.83898
C	-4.75596	1.29395	-3.48309
Rh	-1.38969	0.00475	-0.20153
P	-2.40891	-1.23580	1.45225
C	-1.20674	-1.92279	2.71566
C	-1.24975	-1.57267	4.07933
C	-0.35190	-2.15905	4.98963
C	0.59110	-3.09961	4.55016
C	0.63579	-3.45698	3.19064
C	-0.25495	-2.87017	2.28136
Rh	1.39458	0.05599	0.16029
P	3.25932	1.33976	0.88439
C	4.95519	0.56325	0.99551
C	5.77847	0.45759	-0.14765
C	7.04625	-0.13824	-0.05511
C	7.50620	-0.64321	1.17116
C	6.69389	-0.54176	2.31138
C	5.42844	0.06008	2.22783
Cl	-0.11996	1.49040	1.46306
Cl	0.09455	1.15099	-1.80717
P	2.39134	-1.46013	-1.26818
C	1.18279	-2.25333	-2.45499
C	1.18304	-1.94422	-3.82924
C	0.28678	-2.58896	-4.69981
C	-0.61577	-3.54424	-4.20816
C	-0.62490	-3.85213	-2.83621
C	0.26494	-3.20724	-1.96557
C	3.20947	-2.94941	-0.50307
C	3.73690	-2.87730	0.80135
C	4.39228	-3.98410	1.36468
C	4.52371	-5.17520	0.63328
C	3.99948	-5.25615	-0.66724
C	3.34582	-4.15143	-1.23530
C	3.66719	-0.75094	-2.42779
C	4.79565	-1.49352	-2.83290
C	5.71364	-0.94003	-3.73992
C	5.51513	0.35415	-4.24905
C	4.39086	1.09637	-3.85010
C	3.46963	0.54986	-2.94126
C	3.53135	2.93270	-0.04074
C	4.80244	3.54165	-0.13718
C	4.94160	4.78150	-0.78182
C	3.81934	5.42433	-1.32926
C	2.55287	4.82560	-1.22724
C	2.40481	3.58522	-0.58665
C	2.99502	1.90015	2.64964
C	3.45481	3.16321	3.07536
C	3.32354	3.54709	4.41981
C	2.74106	2.67433	5.35188
C	2.27943	1.41597	4.93254
C	2.39716	1.03176	3.58788
C	-3.28236	-2.79927	0.92732
C	-3.56578	-3.06760	-0.42591
C	-4.22729	-4.25189	-0.79366
C	-4.60820	-5.18060	0.18710
C	-4.31884	-4.92646	1.53856
C	-3.65632	-3.74656	1.90842
C	-3.63370	-0.31391	2.51235
C	-3.21756	0.89840	3.10982
C	-4.07858	1.59215	3.97413
C	-5.36480	1.09391	4.24008
C	-5.78799	-0.09810	3.63297
C	-4.92882	-0.80325	2.77406
C	-2.89404	3.01085	-1.03629
C	-2.86195	3.79399	-2.20782
C	-2.58767	5.16939	-2.12753
C	-2.34489	5.77290	-0.88392
C	-2.36487	4.99388	0.28538
C	-2.62746	3.61796	0.21070
H	3.63690	-1.95368	1.37717
H	4.80108	-3.91123	2.37750
H	5.03128	-6.03883	1.07483
H	4.09532	-6.18211	-1.24330
H	2.93423	-4.22947	-2.24605
H	4.96751	-2.49775	-2.43545
H	6.58771	-1.52373	-4.04666
H	6.23434	0.78280	-4.95440
H	4.22820	2.10573	-4.24108
H	2.59808	1.13269	-2.62869
H	1.88771	-1.20985	-4.22786
H	0.30914	-2.34944	-5.76808
H	-1.30387	-4.05390	-4.89028
H	-1.32249	-4.59701	-2.44043
H	0.25574	-3.46323	-0.90171
H	5.68291	3.05433	0.29109
H	5.93103	5.24506	-0.85184
H	3.93222	6.39084	-1.83118
H	1.67158	5.32109	-1.64684
H	1.41508	3.12488	-0.51440
H	3.91338	3.85222	2.36045
H	3.68088	4.53249	4.73604
H	2.64330	2.97510	6.40006
H	1.81491	0.73093	5.64902
H	2.00282	0.06324	3.26471
H	5.44215	0.85596	-1.10822
H	7.67419	-0.20307	-0.94917
H	8.49660	-1.10449	1.23971
H	7.04896	-0.91785	3.27642
H	4.81770	0.15491	3.13024

H	-3.24831	-2.35668	-1.19567
H	-4.44104	-4.44679	-1.84974
H	-5.12167	-6.10390	-0.09960
H	-4.60322	-5.65110	2.30818
H	-3.42143	-3.56613	2.96231
H	-2.21331	1.28622	2.90920
H	-3.73930	2.52199	4.44271
H	-6.03524	1.63485	4.91559
H	-6.79239	-0.48762	3.82667
H	-5.27352	-1.73051	2.30961
H	-1.98628	-0.85121	4.44170
H	-0.40385	-1.88353	6.04812
H	1.28252	-3.56069	5.26292
H	1.36605	-4.19050	2.83483
H	-0.21263	-3.15666	1.22631
H	-3.04827	3.33530	-3.18245
H	-2.56737	5.76846	-3.04383
H	-2.13698	6.84617	-0.82476
H	-2.16491	5.45445	1.25829
H	-2.61458	3.01430	1.12286
H	-1.72592	-0.29339	-3.22297
H	-2.20805	-0.74777	-5.61566
H	-4.31303	0.11564	-6.66854
H	-5.94002	1.42943	-5.28911
H	-5.47987	1.85883	-2.88775
H	-5.20219	-0.99004	-1.19858
H	-7.54826	-1.24419	-0.43874
H	-8.68708	0.62683	0.78065
H	-7.43604	2.74476	1.24561
H	-5.08980	3.00898	0.48195
H	1.77216	-0.83805	1.35483
H	-1.75979	-1.08948	-1.21686
H	0.00902	-1.03276	0.06648

27. References

(1) Pangborn, A. B.; Giardello, M. A.; Grubbs, R. H.; Rosen, R. K.; Timmers, F. J. *Organometallics* **1996**, *15*, 1518.
(2) Pernik, I.; Hooper, J. F.; Chaplin, A. B.; Weller, A. S.; Willis, M. C. *Acs Catalysis* **2012**, *2*, 2779.
(3) Fryzuk, M. D.; Piers, W. E.; Einstein, F. W. B.; Jones, T. *Canadian Journal of Chemistry-Revue Canadienne De Chimie* **1989**, *67*, 883.
(4) Stephens, F. H.; Baker, R. T.; Matus, M. H.; Grant, D. J.; Dixon, D. A. *Angew. Chem. Int. Ed.* **2007**, *46*, 746.
(5) Dallanegra, R.; Robertson, A. P. M.; Chaplin, A. B.; Manners, I.; Weller, A. S. *Chem. Commun.* **2011**, *47*, 3763.
(6) Jaska, C. A.; Temple, K.; Lough, A. J.; Manners, I. *J. Am. Chem. Soc.* **2003**, *125*, 9424.
(7) Lubben, A. T.; McIndoe, J. S.; Weller, A. S. *Organometallics* **2008**, *27*, 3303.
(8) Kumar, A.; Ishibashi, J. S. A.; Hooper, T. N.; Mikulas, T. C.; Dixon, D. A.; Liu, S.-Y.; Weller, A. S. *Chem. Eur. J.* **2016**, *22*, 310.
(9) Kalviri, H. A.; Gaertner, F.; Ye, G.; Korobkov, I.; Baker, R. T. *Chem. Sci.* **2015**, *6*, 618.
(10) Staubitz, A.; Sloan, M. E.; Robertson, A. P. M.; Friedrich, A.; Schneider, S.; Gates, P. J.; Guenne, J. S. A. D.; Manners, I. *J. Am. Chem. Soc.* **2010**, *132*, 13332.
(11) Ewing, W. C.; Carroll, P. J.; Sneddon, L. G. *Inorg. Chem.* **2013**, *52*, 10690.
(12) Metters, O. J.; Chapman, A. M.; Robertson, A. P. M.; Woodall, C. H.; Gates, P. J.; Wass, D. F.; Manners, I. *Chem. Commun.* **2014**, *50*, 12146.
(13) Dodds, A. R.; Kodama, G. *Inorg. Chem.* **1977**, *16*, 2900.
(14) Rosello-Merino, M.; Lopez-Serrano, J.; Conejero, S. *J. Am. Chem. Soc.* **2013**, *135*, 10910.
(15) Johnson, H. C.; Robertson, A. P. M.; Chaplin, A. B.; Sewell, L. J.; Thompson, A. L.; Haddow, M. F.; Manners, I.; Weller, A. S. *J. Am. Chem. Soc.* **2011**, *133*, 11076.
(16) Espenson, J. H. *Chemical Kinetics and Reaction Mechanisms*; McGraw-Hill, Inc., 1995.
(17) Cosier, J.; Glazer, A. M. *J. App. Cryst.* **1986**, *19*, 105.
(18) Otwinowski, Z.; Minor, W. In *Macromolecular Crystallography, Pt A* 1997; Vol. 276, p 307.
(19) *Oxford Diffraction Ltd, Abingdon, England: 2011.*
(20) Palatinus, L.; Chapuis, G. *Journal of Applied Crystallography* **2007**, *40*, 786.

(21) Betteridge, P. W.; Carruthers, J. R.; Cooper, R. I.; Prout, K.; Watkin, D. J. *Journal of Applied Crystallography* 2003, *36*, 1487.
(22) Spek, A. L. *Journal of Applied Crystallography* 2003, *36*, 7.
